# Networking trait resilience: Unifying fragmented trait resilience systems from an ecological systems theory perspective

**DOI:** 10.1111/jopy.12925

**Published:** 2024-03-01

**Authors:** John Maltby

**Affiliations:** ^1^ School of Psychology and Vision Sciences, College of Life Sciences University of Leicester Leicester UK

**Keywords:** adaptive, network, resilience, systems, trait

## Abstract

**Objective:**

This study reconceptualized trait resilience, defining it as a network of systems; utilizing direct resilience assessments—engineering, ecological, adaptive capacity, social cohesion—and proxy resilience assessments—personality, cognitive, emotional, eudaimonia, and health.

**Background:**

The background of the study addresses the fragmented conceptualization of trait resilience by proposing a unifying network model based on ecological systems theory, illustrating the dynamic interplay of resilience factors across varying levels of disturbance.

**Method:**

In Study One, four USA or UK samples (total *n* = 2396) were used to depict the trait resilience network. Study Two (*n* = 1091) examined the relationship between the network and disturbance at two time‐points, using mental health levels as a disturbance metric.

**Results:**

Study One found that adaptive capacity, and sometimes positive emotional processes, were central variables to the network. Study Two found that in lower disturbance groups, adaptive capacity remained important, while in higher disturbance groups, a broader set of variables became central to the network.

**Conclusions:**

Study One suggests a Broaden‐and‐Build approach, where adaptive capacity is a foundational resilience capability, reciprocally associated with positive emotional mechanisms. Study Two suggests a new “Dynamic Resilience Spectrum Theory,” proposing that increased disturbances necessitate the use of a more diverse set of resilience traits.

## 1 INTRODUCTION

Resilience is crucial in various aspects of human life, including mental health, academic and career success, and personal development (Fletcher & Sarkar, 2013; Liu et al., 2022; Rutter, 2013). While resilience has been extensively studied, its conceptualization remains fragmented, necessitating a unifying framework to reconcile varied interpretations and conceptual approaches. The lack of a clear and consistent definition, particularly regarding essential resilience traits, significantly hampers the development of cohesive and practical theoretical frameworks in academic research (Denckla et al., 2020; Métais et al., 2022). Resilience is frequently reduced to a singular dimension or favored measurement model, thereby neglecting the diversity of skills, strategies, and resources essential for resilient behavior. This may lead to incomplete or skewed interpretations of resilience. Acknowledging individuals demonstrate a range of measurable resilient traits, often in various combinations and contexts, to gain a more nuanced and precise understanding of resilience.

Trait resilience, defined as characteristics that describe how individuals typically perceive and respond to challenges, is evaluated through either “direct” or “proxy” assessments (Maltby & Hall, 2022). Direct assessments focus on standard responses to these types of challenges. Traditional measures like the Hardiness Scale (Bartone et al., 1989) and the Ego‐Resiliency Scale (Block & Kremen, 1996) integrate cognitive, emotional, and behavioral personality characteristics. The Connor–Davidson Resilience Scale (Connor & Davidson, 2003), rooted in clinical psychology, highlights traits like hardiness, goal‐driven strategies, stress tolerance, and faith. Newer scales, like the Predictive 6 Factor Resilience Scale (Rossouw et al., 2017), emphasize resilience's neurobiological aspects. Literature reviews suggest the presence of 40–50 measures to assess characteristics of resilience (Maltby & Hall, 2022; Terrana & Al‐Delaimy, 2023). Recent studies however indicate these measures generally comprise four latent trait factors, aligning with ecological systems theory (Maltby & Hall, 2022). Ecological systems theory (Folke et al., 2004; Gunderson, 2000; Holling, 1996), focusing on ecosystem resilience to disturbances (e.g., disruption affecting an ecosystem's structure or function), identifies four primary systems. First, *Engineering Resilience*, where there is rapid and easy recovery to an original state after disturbances. Second, *Ecological Resilience*, which is the system's capacity to absorb changes during disturbance while retaining its goal‐based functionality. Third, *Adaptive Capacity Resilience*, demonstrating the system's ability to adjust to change and potential for adaptability around disturbance. Fourth, Social‐Ecological Resilience focused on *social cohesion*, portraying the positive integration between social systems, their structures, and ecological settings around disturbance. Despite varied resilience assessments, these four ecological concepts underpin common measures of trait resilience (Maltby et al., 2015; Maltby & Hall, 2022). For example, scales such as the Brief Resilience Scale (Smith et al., 2008), the Composure subscale from the Predictive 6 Factor Resilience Scale (Rossouw et al., 2017), and the Personal Strength/Self subscale from the Resilience Scale for Adults (Friborg et al., 2005) form a latent engineering resilience factor. Also, the Planning Prioritizing Behaviour subscale of the Scale of Protective Factors (Ponce‐Garcia et al., 2015) and the Personal Competence Subscales of the Psychological Resilience Scale (Wagnild & Young, 1993) and the Connor–Davidson Resilience Scale (Connor & Davidson, 2003) form an ecological resilience factors. Thus, direct resilience measures can be summarized into four systems: engineering, ecological, adaptive capacity, and social cohesion.

Proxy assessments of trait resilience explore how different psychological attributes protect against life's adversities, informed by models of stress resilience and buffering techniques (Tronick & Dicorcia, 2015). These studies examine a range of traits to understand how they mitigate the impact of adverse experiences and contribute to resilient outcomes. Therefore, while these traits may not be direct measures of resilience, their contribution to positive outcomes underlines their significance in understanding resilience traits. Maltby and Hall (2022) emphasize five psychological systems that shed light on resilience traits. First is a *Resilient Personality System*. This encompasses positive personality traits that foster resilience, such as emotional stability, extraversion, conscientiousness, openness, agreeableness, and honesty‐humility (Asendorpf & Van Aken, 1999). This system corresponds with the lexical model of personality and can be via the major personality traits. Second, is a *Resilient Cognitive Processing System*. This system integrates theories of resilient functioning, emphasizing executive functions and experiential demand processing (Cicchetti & Curtis, 2015), with a cognitive mapping model that combines current situations, past experiences, and goal‐oriented processes (Parsons et al., 2016). Assessments of this system involve evaluating general cognitive ability and executive functioning. Third, is a *Resilient Emotional Processing System*. This system highlights the link between resilience and effective emotional regulation, underpinned by the Broaden‐and‐Build theory. This theory posits a reciprocal relationship between trait resilience and positive emotional dynamics, like emotional regulation and proactive coping strategies (Kay, 2016; Troy & Mauss, 2011). Measurements of this system focus on emotional regulation and coping abilities. Fourth is an *Eudaimonia Resilience System*. This system reflects the genetic and neural bases of eudaimonic well‐being, emphasizing self‐realization, intrinsic motivation, alignment of actions with values, and authenticity (Ryan & Deci, 2001; Ryff & Keyes, 1995; Waterman et al., 2010; Wright & von Stumm, 2023) with eudaimonic well‐being, acting as a resilient protective factor during significant life changes (Archontaki et al., 2013; Ryff, 2013). Measurement of this system is based on Ryff's well‐being domains (Ryff & Keyes, 1995), that assess autonomy, environmental mastery, purpose in life, personal growth, positive relationships, and self‐acceptance. Finally, a *Resilient Health System*. This system focuses on the interplay between trait resilience and positive health, emphasizing belief in one's ability to manage health challenges (Fredrickson & Branigan, 2005; Rossouw et al., 2017). Assessments of this system involve evaluating general health status. Thus, these five systems offer a comprehensive framework for assessing proxy accounts of resilience traits.

Despite its wealth of data and insights, the field of trait resilience research currently lacks a unifying framework that encompasses its full spectrum of intricacies (Masten et al., 2021). This gap underscores the need to transition from methods focusing primarily on individual traits or preferred multifactor models of resilience to more comprehensive frameworks that capture the complex interplay of factors contributing to trait resilience. Such a holistic approach is essential to understand resilience not just as a personal attribute, but as a dynamic product of various interacting elements. A promising direction in this endeavor is the application of ecological systems theory. This theory, initially formulated to explain the resilience of ecological systems, can be adapted to understand human resilience. It conceptualizes resilience as a multifaceted network of interlinked systems, rather than a series of isolated phenomena (Folke et al., 2004; Gunderson, 2000; Holling, 1996). In ecological terms, trait resilience is the ability of a system to maintain or regain its balance in the face of environmental disturbances that can alter the ecosystem's structure, resource availability, and biodiversity. This perspective shifts the focus from isolated individual traits to a more expansive view, considering the broader array of interactions within the resilience framework. Integrating psychological traits of resilience with ecological systems theory's portrayal of interconnected systems, in which individuals maintain or regain equilibrium in the face of disturbance (e.g., challenges, negative events, or stressors that individuals may experience or encounter), can be used to develop a more comprehensive understanding of human resilience. This approach facilitates a bridge between varied definitions and conceptualizations of trait resilience, paving the way for a cohesive framework. By viewing trait resilience through this ecological lens, a holistic outlook can be gained that merges previously disparate elements and encourages both theoretical and empirical advancements. This integrated perspective could uncover core theories and provide fresh insights into the nature of resilience, examining it as a network of interconnected elements that respond adaptively to different levels of disturbance. Therefore, modeling trait resilience systems akin to ecological systems provides fresh avenues for exploring how these networks withstand and adapt to various challenges.

Against the backdrop of this expansive literature on trait resilience, this study aims to address the current gaps in understanding and integrate various trait resilience frameworks by redefining resilience as a complex network composed of multiple interconnected systems. By leveraging ecological systems theory, this study aims to offer new insights into the dynamic interplay of resilience factors. The study has three objectives, aiming to address the many definitions of trait resilience through the lens of the ecological systems theory:

1. Representation of Trait Resilience Systems as a Network: The initial goal is to construct an empirical framework for trait resilience by charting primary trait resilience systems as a network comprising direct and proxy assessments of resilience systems. This foundational phase is designed to provide a holistic portrayal reminiscent of an ecological system (Study One).
2. Identification of Central Trait Resilience Variables: To identify the central trait resilience systems within the trait resilience networks. By doing so, the objective is to identify which systems are most crucial, thereby refining the understanding of what are the most important trait resilience systems (Study One).
3. Exploration of the Traits Resilience Systems Network in Response to Disturbance: The final objective is to explore the interplay between resilience system networks and varying levels of disturbances. This exploration will shed light on the possible dynamics of the trait resilience network within the larger context of system disturbance (Study Two).

## 2 STUDY ONE

### 2.1 Method

Study One had two objectives: (1) to systematically represent trait resilience networks, and (2) to identify the central variables of trait resilience.

#### 2.1.1 Samples

Data were collected from four distinct samples. This multi‐sample approach ensures that the findings are consistent across diverse groups and not isolated to a single cohort. The samples are as follows: *Sample 1*, 1202 USA adults, sourced from Prolific in 2023; *Sample 2*, 345 UK adults, sourced from Prolific in 2023; *Sample 3*, 426 USA adults from Amazon Mechanical Turk in 2018; and *Sample 4*, 423 UK adults, sourced from Prolific in 2018. There was a focus on the USA and the United Kingdom primarily due to the feasibility of obtaining demographically representative samples from these countries. Prolific was employed to establish the representativeness of Samples 1 and 2, utilizing a recruitment feature that facilitated sampling based on three broad demographic factors: sex/gender, age groups, and ethnicity groups. These are grounded in the 2015 USA census and the 2011 UK census data. Samples 3 and 4 were extracted from a dataset by Maltby and Hall (2022). For research purposes, these samples were segregated into distinct USA and UK respondent clusters. Table 1 provides a detailed demographic breakdown for all samples. Additionally, confidence levels (including the margin of error) are provided to demonstrate how well Samples 1 and 2 mirror the wider USA/UK demographics based on broad assessments of sex/gender, age groups, and ethnicity groups.

**TABLE 1:** Broad demographic characteristics (gender, age [with mean age], and ethnicity) by sample. Representativeness estimates for Samples 1 and 2.

	Sample 1 (USA)	Sample 2 (United Kingdom)	Sample 3 (USA)	Sample 4 (United Kingdom)
	*n* = 1202	*n* = 345	*n* = 426	*n* = 423
Male (*n*=)	583 [585]	177 [177]	216	235
Female (*n*=)	619 [617]	168 [168]	210	188
18–27 years	215 [216]	57 [58]	150	144
28–37 years	212 [210]	57 [56]	164	159
38–47 years	196 [194]	63 [63]	64	72
48–57 years	204 [203]	58 [58]	31	26
58+ years	375 [379]	110 [110]	17	22
Mean age (SD)	45.48 (15.8)	46.15 (16.0)	33.22 (10.5)	33.47 (11.0)
White	931 [936]	290 [291]	332	323
Asian	74 [72]	24 [24]	26	25
Black	153 [153]	10 [10]	26	24
Mixed	24 [24]	11 [10]	30	33
Other	20 [17]	10 [10]	12	18
Representativeness (margin of error)	95%, ±2.83%	95%, ±5.28%		

*Note*: Expected sample numbers for representativeness of Samples 1 and 2 are provided in parentheses. In terms of variation, the overall percentage deviation across all demographic categories for Sample 1 was 0.62% and for Sample 2 was 0.38%.

#### 2.1.2 Measurements

A comprehensive set of assessments was employed to evaluate trait resilience systems, categorizing them as either direct or proxy measurements. This encompassed four direct trait resilience systems—engineering, ecological, adaptive capacity, and social cohesion—and five proxy trait resilience systems—personality, cognitive processing, emotional processing, eudaimonia, and health.

##### Assessment of Direct Trait Resilience

A range of scales was used to directly assess trait resilience, covering the four established trait resilience systems. Given the myriad of scales potentially indicative of each system, Principal Components Analysis (PCA) was employed for Samples 1, 3, and 4. This analysis was intended to yield a factor score from the primary component, as elaborated in Supporting Information S2. The approach was based on using scales recommended by Maltby and Hall (2022) due to their significant loadings in previous factor analyses. In contrast, for Sample 2, distinct trait resilience scales were used to assess each system. Collectively, four direct trait resilience systems were assessed, which are outlined below.

###### Engineering Resilience

In Samples 1, 3, and 4, a factor score was derived from scores from the Brief Resilience Scale (Smith et al., 2008), the Composure subscale of the Predictive 6 Factor Resilience Scale (Rossouw et al., 2017), and the Engineering Resilience subscale of the Resilient Systems Scales (Maltby et al., 2019). For Sample 2, this dimension was assessed using only the Engineering Resilience Subscale of the Resilient Systems Scales (Maltby et al., 2019). In Samples 1 and 2, two items on this subscale were revised to exclude the terms “normal self” and the colloquialism “get‐back.”

###### Ecological Resilience

In Samples 1, 3, and 4, a factor score was derived from scores from the Planning Prioritizing Behaviour Subscale (Ponce‐Garcia et al., 2015), the Personal Competence Subscale (Wagnild & Young, 1993), and the Structured Style Subscale of the Resilience Scale for Adults (Friborg et al., 2005). Sample 2 utilized the Ecological Resilience Subscale from the Resilient Systems Scales (Maltby et al., 2019).

###### Adaptive Capacity

Samples 1, 3, and 4, a factor score was derived from scores from the Social Competence Subscale of the Resilience Scale for Adults (Friborg et al., 2005), the Adaptive‐Capacity Subscale of the Resilient Systems Scales (Maltby et al., 2019), and the Social Skills Subscale of the Scale of Protective Factors (Ponce‐Garcia et al., 2015). In Sample 2, the Adaptive Capacity subscale of the Resilient Systems Scales (Maltby et al., 2019) was used.

###### Social Cohesion

In Samples 1, 3, and 4, a factor score was derived from scores from the Psychological Care subscale of the Adult Resilience Measure (Resilience Research Centre, 2013; Ungar & Liebenberg, 2011), the Family Cohesion subscale of the Resilience Scale for Adults (Friborg et al., 2005), and the Social Support Subscale from the Scale of Protective Factors (Ponce‐Garcia et al., 2015). For Sample 2, to fill a gap in a brief resilience assessment to assess social cohesion resilience (Maltby & Hall, 2022), and align with the shorter measures used to measure the other resilience dimension four new items was crafted to assess Social Cohesion resilience, with descriptions capturing feelings of resilience in terms of secure social ties, shared values, and supportive relationships (“I have people in my life … (i) who care about me, (ii) who support me, (iii) with whom I share a sense of unity, and (iv) and we take care of each other”). Findings that support the reliability and validity of this scale are provided in Supplementary Material S1.

##### Proxy Assessments of Trait Resilience

Five proxy resilience systems were assessed across the four samples. This assessment was consistent across the samples, with exceptions noted.

###### Resilient Personality System

Personality was assessed via two measures—(i) the 10‐Item Personality Inventory (Gosling et al., 2003) evaluating Extraversion, Emotional Stability, Conscientiousness, Agreeableness, and Openness, and (ii) the Brief HEXACO (De Vries, 2013) inventory's Honesty‐humility subscale appraising sincerity, fairness, greed avoidance, and modesty traits.

###### Cognitive Processing System

Cognitive Processing involved assessing two distinct variables: cognitive ability and executive functioning. Cognitive ability was gauged using Set 1 of the Raven's Progressive Matrices (Harcourt Assessment, 2003; Raven et al., 1998), while executive functioning was assessed either by the 6‐item Webexec tool (Buchanan et al., 2010) for Samples 1 and 2, or the 20‐item Dysexecutive Functioning Questionnaire (Wilson et al., 1998) for Samples 3 and 4.

###### Emotional Processing System

Emotional processing was assessed via a factor score, based on findings by Maltby and Hall (2022), via the subscales of the Emotional Regulation Questionnaire (Gross & John, 2003) and the Coping Strategies Inventory Short‐Form (Jackson Heart Study, 2000). Further details on the loadings across the samples are provided Supplementary Material 2, with the factor score representing a continuum of cognitive appraisal and engagement coping versus expressive suppression and disengagement coping.

###### Eudaimonia Resilience System

Eudaimonia was assessed via total scores on the Mental Health Continuum Short Form (Keyes, 2009) among all four samples comprising assessments of Autonomy, Environmental Mastery, Positive Relationships with Others, Personal Growth, Purpose in Life, and Self‐acceptance.

###### Resilient Health System

Health was assessed using the 1‐item self‐rated health (SRH) question (“Would you say your health in general is…”) (Idler & Benyamini, 1997), with responses ranging from “Excellent” to “Poor” (This variable was not reported in the original Maltby & Hall, 2022 study). The 1‐item self‐rated health question is deemed a robust health measure due to its proven validity and reliability in predicting morbidity and mortality across diverse groups (DeSalvo et al., 2005; Jylhä, 2009).

All scales were administered via an online survey platform. To counteract potential order effects, the presentation of measures was randomized for Samples 1 and 2. However, Samples 3 and 4 adhered to a predetermined sequence based on the dataset provided by Maltby and Hall (2022).

#### 2.1.3 Analysis strategy

Network analysis was conducted using both JASP and R, employing the *bootnet, qgraph*, and *networktools* packages in R (Epskamp et al., 2012, 2018). In this analysis, trait resilience systems were illustrated as nodes, while their interconnections were represented as edges. The analysis for Study One was organized into three main stages: Stages 1 and 2 focused on the first objective, and Stage 3 targeted the second objective of the study.

- Stage 1: Network Analysis and Centrality. In this stage, the structure of the resilience network was charted by identifying central nodes, which are essential components within the network. This step is fundamental to understanding which trait resilience systems play significant roles in the overall network's composition.
- Stage 2: Network and Edge‐weight Stability. Stage 2 provided assessments of Network and Edge‐weight Stability that builds on the initial analysis by evaluating centrality stability and edge weight using bootstrap methods. This stage aims to gauge the robustness and reliability of the centrality indices, and to establish the stability of the relationships between nodes (representing trait resilience systems) and edges (indicating trait resilience systems correlations).
- Stage 3: Strength centrality and bootstrapped centrality difference test. This examines core trait resilience processes, utilizing strength centrality statistics and the bootstrapped centrality difference test. This stage aims to identify significant disparities in strength centrality among nodes, highlighting core network variables crucial for network trait resilience, and thereby understanding the mechanisms underpinning the network's trait resilience.

All the data samples of *n* ≥ 250 are considered adequate for establishing adequate sensitivity, specificity, and edge weights correlation, for network analyses encompassing 20 or fewer variables (Constantin, 2019).

### 2.2 Results

#### 2.2.1 Objective 1: Systematic representation of trait resilience networks

To ensure the robustness and generalizability of the findings, separate network analyses were conducted for each of the four samples, emphasizing the replicability and consistency of the results. This approach demonstrated that the identified network patterns of trait resilience were consistent across different samples and not merely time‐specific phenomena. Such an approach is crucial in network analysis, especially when investigating constructs like resilience, which may manifest differently across populations and over time. Replicating these findings across diverse samples and time periods allows the assessment of the stability and consistency of resilience traits and their interconnections, reinforcing the network model's validity.

##### Network Estimation

**FIGURE 1:**
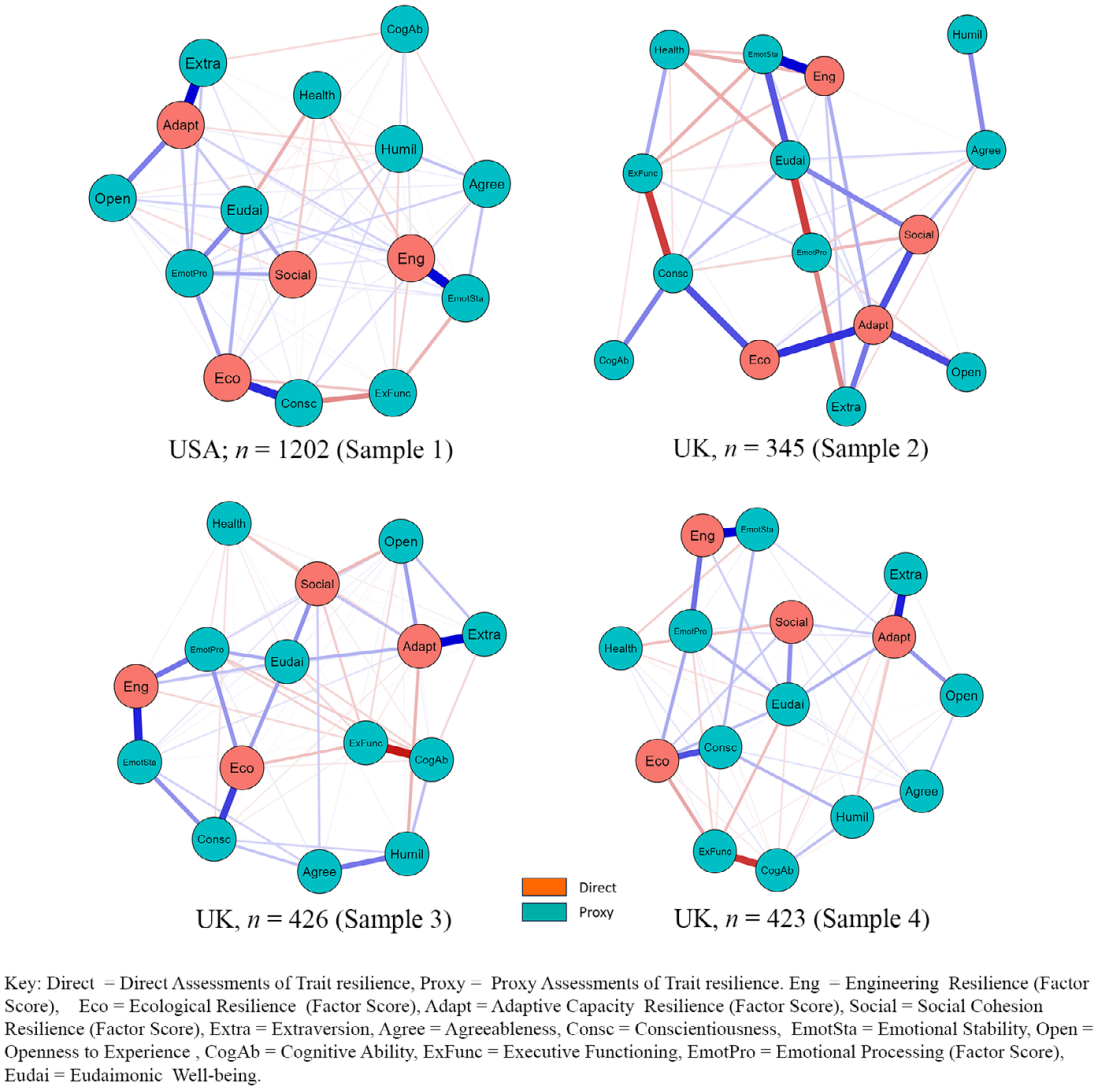
Illustrations of the trait resilience network systems among four samples.

The analysis assessed the strength of associations between nodes. The EBICglasso method was used with the LASSO regularization in the Gaussian graphical model to shrink weak associations toward zero (and some to zero) to create a simpler model. A tuning parameter of 0.5 simplified the model, removing potential false‐positive edges. See Figure 1 for the findings.

##### Centrality Assessments

The centrality assessment comprised the computation of Strength, Betweenness, and Closeness centrality indices for each node within the network. Strength centrality represents the sum of the weights of all links connected to a node, indicating its level of connectivity. Betweenness centrality represented the frequency a node serves as a bridge on the shortest path between two other nodes. Closeness centrality gauged a node's proximity to all other nodes within the network. The normalized (z‐scored) values of these indices were charted for each node to evaluate their significance within the network, as showcased in Table 2. Adaptive capacity displayed the highest value for Strength centrality across all samples, highest values for Betweenness centrality in Samples 2–4, and highest value for Closeness centrality in Sample 2. Emotional processing scored the highest for Betweenness and Closeness centrality in Sample 1, while Eudaimonia scores were highest for Closeness centrality in Samples 3 and 4.

**TABLE 2:** Between, closeness and strength centrality statistics of the Resilient Systems Network by sample.

	Betweenness				Closeness				Strength			
	Sample											
	1	2	3	4	1	2	3	4	1	2	3	4
Engineering	0.64	−0.43	0.34	−0.54	0.56	−0.10	−0.23	−0.17	0.90	0.30	0.32	0.51
Ecological	0.95	0.65	0.53	1.05	0.63	0.81	1.25	1.35	0.83	−0.13	0.53	0.79
Adaptive capacity	1.43	**2.37**	**2.25**	**2.24**	0.76	**1.55**	0.58	0.51	**1.53**	**1.73**	**2.05**	**1.64**
Social cohesion	−0.80	1.29	−0.04	−0.14	−0.24	1.16	0.97	0.43	−0.18	0.06	0.23	−0.41
Extraversion	−0.32	−0.70	−0.80	−1.07	0.07	0.08	−0.27	−0.47	−0.16	−0.56	−0.64	−0.54
Agreeableness	−0.80	0.20	−0.61	−1.07	−0.65	−1.32	−0.31	−1.64	−0.96	−0.50	−1.22	−1.19
Conscientiousness	0.64	0.92	−0.04	0.52	0.47	0.47	0.72	0.64	0.22	1.02	0.20	0.24
Emotional stability	0.16	−0.07	−0.61	0.26	0.33	0.20	−0.62	0.04	0.63	0.87	−0.06	0.30
Openness	−1.11	−0.97	−1.18	−0.80	−0.72	−0.12	−1.11	−1.25	−0.62	−1.12	−0.74	−1.66
Humility‐honesty	−0.64	−0.97	−0.23	−0.14	−0.86	−2.13	−0.29	−0.15	−0.78	−1.65	−1.07	−0.85
Cognitive ability	−1.11	−0.97	−0.23	−0.80	−2.36	−1.11	−0.34	−0.38	−1.88	−1.47	0.25	0.37
Executive functioning	−0.48	−0.34	−0.23	0.12	−0.29	−0.19	−0.25	0.24	−0.47	0.24	0.78	0.48
Emotional processing	**2.07**	−0.70	−0.04	−0.14	**1.57**	0.35	0.66	0.57	1.19	0.53	0.57	0.41
Eudaimonia	0.48	0.65	2.06	1.58	1.43	1.06	**1.61**	**1.90**	1.00	1.30	0.81	1.44
Health	−1.11	−0.97	−1.18	−1.07	−0.70	−0.72	−2.36	−1.64	−1.25	−0.62	−2.02	−1.53

*Note*: In each sample, the values highlighted in bold indicate the variable that possesses the highest centrality statistic for the network.

##### Network Stability Assessment

The centrality and edge‐weight stability were assessed using two bootstrap methods: Case and nonparametric bootstrapping, with further details provided in Supplementary Material Figures S3–S6.

Case bootstrap retained the network structure while resampling entire cases (nodes or edges) and was applied to evaluate edge weights and centrality indices stability. Nonparametric bootstrapping (1000 resamples) was conducted to compute a 95% confidence level. As more cases were dropped, edge weights and centrality measures were reevaluated, resulting in a centrality stability coefficient for each measure. These analyses are detailed in Supplementary Material Figure S3. The stability coefficients for edge weights and betweenness, closeness, and strength centrality measures were ≥0.50, meeting the predetermined criteria of being preferably above 0.5 and not below 0.25, and indicating reliable and accurate network analysis estimates (Epskamp et al., 2018).

**FIGURE 2:**
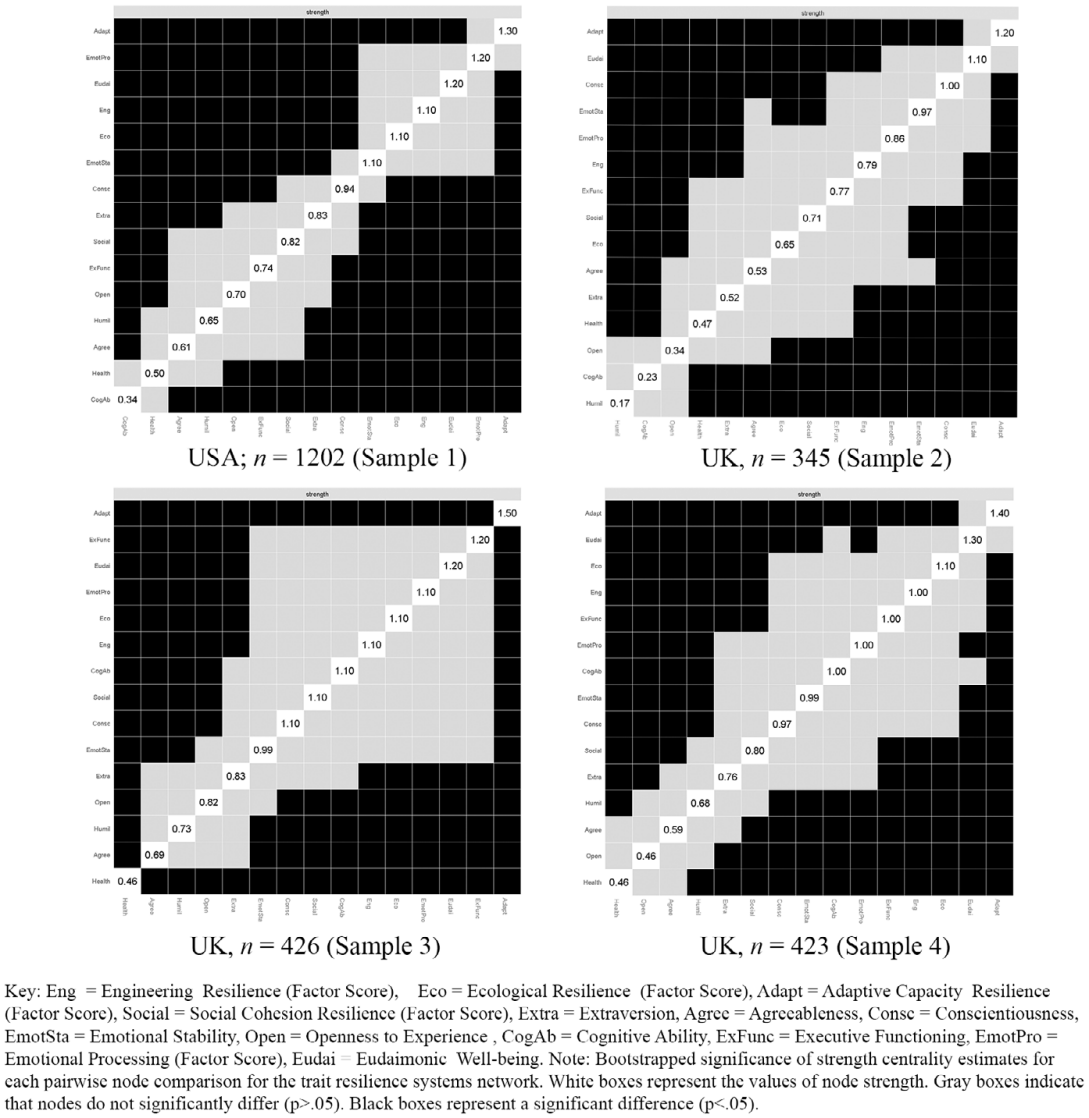
Bootstrapped centrality difference test for strength centrality statistics.

Information is also provided for edge weight stability and node strength using nonparametric bootstrapping that encompassed two calculation sets. Firstly, to gauge edge weight accuracy, nonparametric bootstrapping (1000 replications) was conducted to establish 95% confidence intervals of edge values, as depicted in Supplementary Material Figure S4. Secondly, bootstrapped difference tests (1000 replications, 95% confidence) were performed to discern significant disparities between edge weights strengths (*Bootstrapped edge weights difference test*) and node strengths (*Bootstrapped centrality difference tests*). The analysis of how the edge weights strengths varied across different bootstrap samples are illustrated in Supplementary Material Figure S5. The specific analysis of node strengths for betweenness and closeness centrality statistics is provided in Supplementary Material Figure S6. The Strength centrality statistics, which are a primary focus for Objective 2, are presented in Figure 2.

#### 2.2.2 Objective 2: Identification of central trait resilience variables

The second part of the study assessed core trait resilience processes using strength centrality statistics and the bootstrapped centrality difference test for strength centrality statistics across the four samples (See Figure 2). Strength centrality provides a more relevant measure for assessing important variables within a trait resilience systems network. A node with high strength centrality indicates a variable with significant influence or interaction with other systems, proportional to its importance or influence in a trait resilience system, which is consistent with the approach of identifying the central systems in the trait resilience systems network. Closeness (assessing proximity) and betweenness (acting as a bridge) centrality measures do not translate as readily to such an interpretation in the trait resilience context as neither produces a comprehensive view of a node's true influential capacity or its role in ensuring network resilience.

The bootstrapped centrality difference test is a statistical method used to ascertain whether the centrality of one node significantly differs from others, thereby identifying core network variables. This test compares the centrality measures of different nodes and provides a statistical basis for identifying nodes that hold a central position within the network. Among the samples, the adaptive capacity node consistently scored the highest. However, the bootstrapped centrality difference test showed adaptive capacity scores were not significantly different from emotional processing in Sample 1 or from Eudaimonia in Samples 2 and 4.

### 2.3 Discussion

In Study One, adaptive capacity was identified as a central trait within the resilience network. Moreover, the emotional processing and eudaimonia systems occasionally emerged as central within this network. This supports the “Broaden‐and‐Build” theory, indicating that adaptive capacity, reciprocally with associated positive emotional dynamics like emotional regulation, proactive coping, and eudaimonia, plays the most important role in the trait resilience network.

## 3 STUDY TWO

### 3.1 Method

Study Two explores the interplay between resilience system networks and varying levels of disturbances, as outlined in Objective 3.

#### 3.1.1 Sample

Out of the 1202 participants from the representative USA sample in Study One, 1091 (90.76%) completed a Time 2 segment of the study, 2 weeks after the administration of Time 1. The sample's demographic breakdown, juxtaposed against expected figures in brackets, is as follows: 535 [531] males, 556 [560] females; five age brackets—18–27 years (*n* = 188 [194]), 28–37 years (*n* = 191 [190]), 38–47 years (*n* = 182 [176]), 48–57 years (*n* = 183 [186]), and 58+ years (*n* = 347 [345]); and five ethnic groups—White (*n* = 841 [849]), Asian (*n* = 66 [66]), Black (*n* = 142 [140]), Mixed (*n* = 23 [20]), and Other (*n* = 19 [16]). The total deviation between the expected and actual figures across all demographics was 1.37%. Despite this slight variation, with a confidence level of 95% and a margin of error of ±2.97%, this sample robustly reflects the broader USA population based on these general demographic parameters.

#### 3.1.2 Design

The research approach, named “Resilience Network Mapping” is focused on depicting the relationship between changes in network structures and various levels of disturbances in human systems. In this context, “disturbance” is broadly understood as any range of challenges, negative events, or stressors that individuals might experience or encounter. This approach, inspired by the study of intricate systems like ecological systems, delves into the relationship between a system's structural layout and system disturbance. This analysis encompasses:

- Examination of potential network structures and key processes at the initial time point to understand how specific networks are associated with levels of disturbance.
- Comparison of network structures and key processes across a subsequent incidence of disturbance to assess whether similar disturbances yield consistent network configurations in terms of structure and key processes.

This methodology operates on the hypothesis that the initial network configuration encapsulates vital information (in the current case, central resilience traits) that unveil associations with disturbance levels. This approach helps us discern whether different disturbance levels correspond to different network structures.

Collecting data at two time points introduces a temporal dimension to the analysis, enhancing the understanding of trait resilience systems and their interaction with disturbance. This step aids in replicating the findings from the initial time point, providing a foundation to observe whether the initially identified associations and configurations persist, illustrating that the associations observed are not mere coincidences or isolated occurrences.

#### 3.1.3 Assessment

The study employed hedonic mental health indicators for assessing disturbance levels. Hedonic mental health indicators evaluate short‐term emotional well‐being, primarily focusing on the presence of positive emotions and the absence of negative emotions. Thus, they differ theoretically and empirically from eudaimonic well‐being indicators (Keyes et al., 2002; Linley et al., 2009), which focus on meaningful life fulfillment, as used in the current resilience network. Hedonic indicators play a vital role in evaluating immediate emotional states and accurately capturing temporal fluctuations at specific time points.

Additionally, mental health exists on a continuum, yet the application of clinical cutoffs offers distinct advantages. These thresholds create clear categories, sharpening distinctions and streamlining the recognition of patterns pivotal for defining and addressing mental health issues (Beck et al., 1996; Spitzer et al., 2006). Such categorization is also useful when emulating varying disturbance levels in the model. It substantially enhances the ability to contrast specific trait resilience patterns in relation to both low and high disturbances, rooted in established and substantive benchmarks.

In alignment with the previously described Resilience Network Mapping approach, mental health was evaluated at two separate time points.

##### Time 1

Both direct and proxy trait resilience systems were assessed. The direct systems included engineering, ecological, adaptive capacity, and social cohesion, while the proxy systems consisted of personality, cognitive processing, emotional processing, eudaimonia, and health as elaborated in Study 1. For the factor score evaluations, scores were recalculated using data from Sample 1. Mental health was assessed using the four anxiety or depression items from the PHQ–4 (Kroenke et al., 2009), with responses referencing the past 2 weeks. This scale has a potential score range of 0 to 12. Participants scoring ≥6, indicating at least a “moderate” clinical case, were classified into the “higher disturbance” category. Those scoring below 6 were grouped into the “lower disturbance” category.

##### Time 2

The mental health assessment using the PHQ‐4 was administered again 2 weeks later. Scores were again allocated into further lower and higher disturbance groups using the ≥6 cutoff.

#### 3.1.4 Data analysis

Participants were categorized into “higher” and “lower” disturbance groups at both time points, based on the defined clinical cutoffs of poorer (higher disturbance) and better (lower disturbance) mental health, resulting in four distinct subsamples. Similar to Study One, Network Estimation, Centrality Assessments, and Stability Analysis were used to investigate the trait resilience networks in the four subsamples. Network properties between the two mental health groups at both time points were compared using the permutation‐based Network Comparison Test (NCT), focusing on network invariance and global strength. The detailed statistical assumptions and results for the NCT, including permutation parameters, are presented in the Supplementary Information. Centrality statistics were used to discern levels of betweenness, closeness, and strength centrality across subsamples. This targeted key trait resilience processes using strength centrality statistics and bootstrapped centrality difference tests.

### 3.2 Results

#### 3.2.1 Objective 3. Exploration of trait resilience network behavior amidst disturbance

##### Subsample Composition

Four distinct samples were crafted based on the level of mental health categorized as “higher” and “lower” disturbance across two timepoints. At Time 1, the higher disturbance group (poorer mental health) comprised 344 individuals (31.5%), and the lower disturbance group (better mental health) included 747 individuals (68.5%). At Time 2, the higher disturbance group (poorer mental health) comprised 337 individuals (30.9%), and 754 individuals (69.1%) were categorized under a lower disturbance group. Notably, a significant transition of 18.8% in the mental health sample between the higher and lower disturbance groups was recorded between Time 1 and Time 2, reflecting a fluctuating environment. As observed in Study One, the sample adequacy (*n* ≥ 250) for conducting network analysis on 20 or fewer variables was confirmed (Constantin, 2019). Additionally, to demonstrate replication of the prevalence of mental health levels in this sample, a demographically representative (gender, ethnicity, and age) UK Prolific sample (*n* = 626) was collected at the same time and a similar 30.8% rate was observed among those who would be categorized in the higher disturbance group.

##### Network Estimation, Centrality Assessments, and Stability Analysis

Network analysis was conducted on trait resilience systems for the two mental health groups across both time points. Supplementary Material S7 displays the trait resilience networks, and centrality statistics are detailed in Table 3. Stability coefficients achieved values above 0.50, suggesting a robust network. Further stability details can be viewed in Supplementary Information S8. The correlation stability coefficients for edge weights and betweenness, closeness, and strength centrality measures achieved a value of ≥0.50, satisfying the stipulated criteria of being preferably above 0.5 and never below 0.25. Bootstrapped edge weights, difference tests, and centrality difference tests for betweenness and closeness nodes are further elaborated in the Supplementary Information S9–S11.

**TABLE 3:** Between, Closeness and Strength centrality statistics of the Resilient Systems Network by Time Point and Disturbance Level.

	Betweenness				Closeness				Strength			
	Sample											
	Time 1		Time 2		Time 1		Time 2		Time 1		Time 2	
	Lower	Higher	Lower	Higher	Lower	Higher	Lower	Higher	Lower	Higher	Lower	Higher
Engineering	−0.09	**1.82**	0.04	**2.09**	−0.07	0.83	−0.20	0.96	0.66	0.66	0.45	0.93
Ecological	1.32	0.58	0.91	0.68	0.98	0.63	0.92	0.51	0.98	1.04	0.95	0.96
Adaptive capacity	0.80	1.45	**1.77**	1.57	0.73	0.88	0.55	1.16	**1.68**	1.17	**1.71**	1.19
Social cohesion	−0.61	−0.04	−0.58	−0.98	0.01	−0.30	0.43	−0.89	−0.14	−0.24	0.12	−0.44
Extraversion	0.03	−0.54	−0.58	−0.60	0.52	0.28	0.01	0.67	−0.01	−0.03	−0.17	−0.08
Agreeableness	−0.99	−0.29	−0.95	−0.34	−0.55	−0.80	−0.69	−0.56	−1.19	−0.99	−1.24	−0.85
Conscientiousness	1.32	0.08	0.66	0.04	0.70	0.44	0.70	0.19	0.30	0.70	0.35	0.48
Emotional stability	−0.22	0.95	−0.21	0.17	−0.13	0.39	−0.30	0.21	0.53	0.19	0.52	0.25
Openness	−0.99	−1.16	−0.95	−0.98	−0.47	−0.30	−0.39	−0.07	−0.61	−0.56	−0.47	−0.85
Humility‐honesty	0.99	−1.16	−0.95	−0.72	−0.92	−1.73	−0.77	−1.42	−1.07	−1.43	−1.04	−1.08
Cognitive ability	−0.99	−1.16	−0.95	−0.98	−2.29	−2.21	−2.35	−2.26	−1.71	−1.68	−1.86	−1.64
Executive functioning	−0.09	−0.29	−0.21	−0.34	−0.20	−0.15	−0.13	−0.14	−0.31	−0.50	−0.29	−0.48
Emotional processing	**2.09**	1.20	1.65	0.04	**1.48**	**1.28**	**1.59**	0.88	1.15	**1.31**	1.10	1.30
Eudaimonia	0.42	−0.29	1.28	1.32	1.30	1.11	1.45	**1.28**	0.88	1.27	0.92	**1.39**
Health	−0.99	−1.16	−0.95	−0.98	−1.11	−0.37	−0.81	−0.54	−1.15	−0.91	−1.05	−1.09

*Note*: In each sample, the values highlighted in bold indicate the variable that possesses the highest centrality statistic for the network.

###### Network Comparisons

The Network Comparison Test (NCT) from the R package *NetworkComparisonTest* (van Borkulo et al., 2022) was used to discern any disparities between the two mental health groups from the first and second surveys. The NCT is a robust permutation‐based method that allows us to scrutinize the differences between two networks, focusing on two key aspects: network invariance and global strength. The network invariance test evaluates whether the connections between the same nodes differ, while the global strength test assesses whether the overall strength of all connections, sometimes referred to as global strength, varies between the networks (van Borkulo et al., 2022). NCTs were run 1000 times, with significant differences between the two networks estimated using a *p* < 0.05 (two‐tailed) level of significance. No significant differences were found in the network structures of lower and higher mental health groups at either time point. (Time 1, *M* = 0.16, *p* = 0.09; Time 2, *M* = 0.13, *p* = 0.39) and no significant difference in global strength (Time 1, *S* = 0.33, *p* = 0.57; Time 2, *S* = 0.44, *p* = 0.43). The analysis did not yield significant evidence of differences in network structure, connections, or relationship intensity between the two mental health groups across both time points, suggesting consistent connectivity patterns within the networks.

###### Centrality Statistics Description

Table 3 displays betweenness, closeness, and strength centrality statistics for the sample. Adaptive capacity showed the highest strength centrality amid lower disturbance at both Time 1 and Time 2 and achieved the highest betweenness centrality amid lower disturbance at Time 2. Emotional processing scored highest for betweenness centrality in the lower disturbance sample, was highest for closeness centrality for both disturbance levels at Time 1 and lower disturbance levels at Time 2 and was highest for strength centrality for higher disturbance at Time 2. Eudaimonia was highest for closeness and strength centrality amid higher disturbance at Time 2. Engineering resilience was highest for closeness and strength centrality amid higher disturbance for both Time 1 and Time 2.

###### Central Strength Factors

**FIGURE 3:**
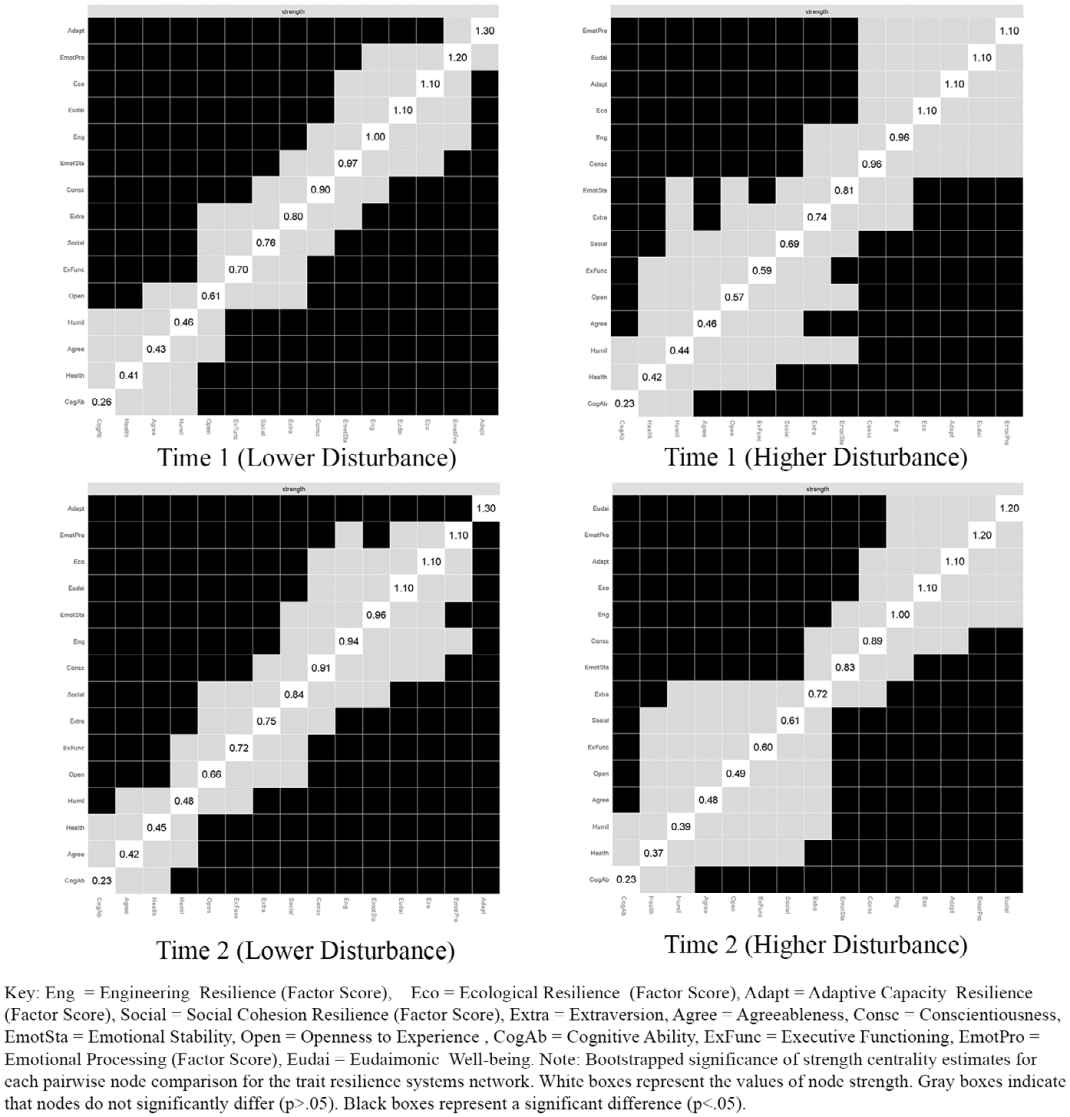
Bootstrapped centrality difference test for strength centrality statistics for each time point by disturbance level.

Strength centrality statistics and bootstrapped centrality difference tests (as illustrated in Figure 3) were used to identify the core mechanisms within the trait resilience networks. The methodology from Study One was adopted, focusing on high strength centrality statistics to deeply investigate the central elements of the network. The aim was to statistically identify significant variances in the strength centrality of one node compared to others, thus pinpointing the core network variables. In the group with lower disturbance, the attribute adaptive capacity showed the highest strength statistics at both observed time points. The centrality statistics were found only to be not significantly different from emotional processing at Time 1. Conversely, in the group with higher disturbance, emotional processing and eudaimonia emerged with the highest strength statistics at Time 1 and Time 2, respectively. However, several nonsignificant differences were observed with conscientiousness, engineering resilience, ecological resilience, and adaptive capacity and eudaimonia not being significantly different from emotional processing at Time 1, and engineering resilience, ecological resilience, adaptive capacity, and emotional processing not being significantly different from eudaimonia at Time 2.

### 3.3 Discussion

Study Two emphasizes the dynamic nature of resilience trait systems in responding to varying disturbance levels. Contrasting with Study One, where adaptive capacity was central, and Study Two reveals an interplay of resilience traits across various mental health disturbance levels. This study shows that adaptive capacity is key at lower disturbance levels, while an expanded range of resilience systems gains importance in the network under higher disturbance levels.

## 4 GENERAL DISCUSSION

This study suggests a shift in understanding trait resilience, envisioning it as a complex, interconnected network, offering new insights into its multifaceted nature.

Study One aimed to delineate primary resilience systems and identify the central variables within this network across four samples collected in the United Kingdom and USA, spanning a 5‐year interval. Notably, two of these datasets closely mirrored the demographic composition of their respective populations in terms of gender, age, and ethnicity. The network structure unveiled in Study One aligns with the mixed model of trait resilience as outlined by Maltby and Hall (2022). This provides valuable clarity regarding the connections between distinct trait resilience types and broader psychological traits often regarded as facilitators of resilience. For instance, are the positive correlations between engineering resilience and emotional stability, ecological resilience and conscientiousness, and adaptive capacity resilience with both extraversion and openness. These insights underscore a congruence, both in theory and empirically, between direct and proxy assessments of trait resilience. Furthermore, they underscore the significance of interpreting trait resilience through the lens of systems theory. The evident interconnections draw parallels with the interdependencies observed in natural ecological systems (Folke et al., 2004; Holling, 1996), accentuating the importance of encompassing the relationships between direct and proxy factors in the analysis of trait resilience to capture the full range of trait resilience systems.

In Study One, an analytical approach was employed across four samples to determine the central variables within the trait resilience systems network. Strength centrality was used to establish the network's most influential nodes, thereby offering an understanding of the predominant resilience traits within the system's dynamics. Adaptive capacity consistently emerged as the predominant node in terms of strength centrality across the samples, affirming its central role within the trait resilience network. Adaptive capacity, a foundational concept in resilience theory, particularly from an ecological systems perspective (Gunderson, 2000), possessing the capacity to navigate change, engage with diversity, demonstrate flexibility, and serve as a safeguard against potential uncertainties, appears to be the most influential system in the trait resilience systems network. While the strength centrality of adaptive capacity was evident, it did not significantly surpass the strength of emotional processing in Sample 1 or eudaimonia in Samples 2 and 3. This focus on positive emotional trait dynamics, such as eudaimonic well‐being, emotional regulation, and positive coping strategies, suggests alignment with the Broaden‐and‐Build perspective, which posits a reciprocal relationship between resilience and positive emotional processes (Kay, 2016; Troy & Mauss, 2011). These findings suggest that adaptive capacity plays a central role in facilitating individuals in successfully navigating challenging situations via the adoption of effective positive emotional processes, including cognitive appraisal, positive coping, and eudaimonia. However, observed variations in resilience traits, such as eudaimonia, across diverse samples merit attention. Nonetheless, distinct patterns linking these variations to specific countries, timeframes, or assessment methods remain unclear. This ambiguity points to the potential role of numerous interconnected factors in contributing to these differences. It is important to note in large‐scale studies involving diverse populations, degrees of variability are expected. The results necessitate a call for further investigation into the factors influencing resilience traits, leading to more detailed future research in this area.

**FIGURE 4:**
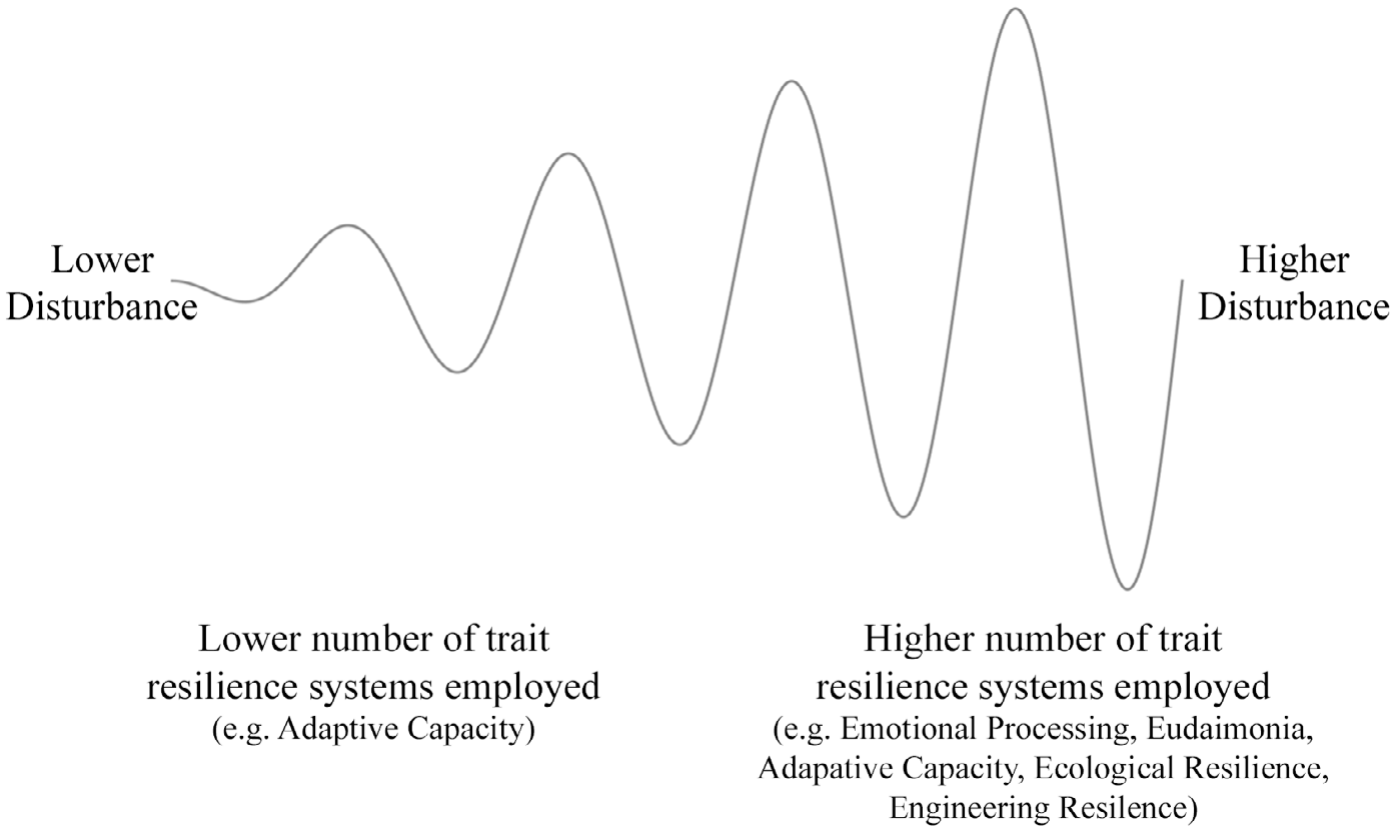
Illustration of the Dynamic Resilience Spectrum Theory.

Building on the findings from Study One, Study Two delved deeper into the resilience system networks. It employed a novel method to chart these networks across different levels of disturbances, examining them at two distinct time points. Based on their mental health status at two distinct time points, participants were grouped into “higher” and “lower” disturbance categories. Notably, consistent node connection patterns emerged across these groups, unaffected by the level of disturbance suggesting a similar underlying structure to the trait resilience networks in lower and higher disturbance. For the lower disturbance group, the findings aligned with those of Study One, pinpointing adaptive capacity as the key central variable in the trait resilience network. In contrast, the higher disturbance group exhibited a more varied centrality pattern. At Time 1, emotional processing demonstrated the highest centrality, while at Time 2, eudaimonia was the highest. Other variables, including engineering resilience, ecological resilience, adaptive capacity (both time points), conscientiousness, and eudaimonia (Time 1), as well as emotional processing (Time 2), were demonstrated as important to the network. These observed differences between the low and high disturbance groups cannot be dismissed due to small sample sizes causing unstable results. This view is reinforced by all samples meeting adequate size standards, meeting the criteria for demonstrating network stability, demonstrating similar structures from the Network Comparison analysis, and the sample size of the high disturbance group being of a similar sample size to one of the UK samples in Study One. These observations introduce the Dynamic Resilience Spectrum Theory, diverging from the “Broaden‐and‐Build” interpretation initially inferred from Study One's findings. This theory posits that individuals diversify their reliance on resilience systems, tapping into a broader set of resilience traits during heightened disturbances (See Figure 4), a concept not previously delineated in existing literature. Contrary to traditional models that perceive resilience as a singular set of traits, the Dynamic Resilience Spectrum Theory envisions resilience as a dynamic spectrum, adapting to levels of mental health. This theory aligns with ecological systems theory, suggesting that just as ecosystems adapt to environmental changes, individuals adjust their resilience strategies according to the severity of challenges faced (Folke et al., 2004; Gunderson, 2000; Holling, 1996). The central premise of the Dynamic Resilience Spectrum Theory is its emphasis on the fluidity of resilience traits, rather than a fixed set of characteristics. Bridging the gap between disparate resilience definitions, the Dynamic Resilience Spectrum Theory proposes a holistic network perspective, revealing resilience as a dynamic interplay of multiple factors rather than a singular set of characteristics. This nuanced understanding has substantial potential for reflecting on and developing mental health support and educational and policy programs around resilience, suggesting a more holistic resilience‐building approach that could transform support strategies for individuals facing challenges across several domains.

Nonetheless, this study has limitations. The absence of differentiation between various types and durations of disturbances might lead to an oversimplified understanding of resilience mechanisms. Different disturbances, varying in nature and length, likely elicit distinct resilience responses, a nuance not captured in this analysis. Secondly, the brief 2‐week interval between assessments constrains the depth of insight into long‐term resilience strategies, possibly neglecting their evolution and sustainability over time. Third, the study did not comprehensively explore resilience variability across different individuals and contexts. Further research, encompassing diverse populations and considering individual variances like demographic factors and personal histories, alongside different environmental contexts, is crucial for a nuanced understanding of resilience. Lastly, employing structural equation modeling and cross‐lagged panel models could yield further insights into the data's temporal dynamics. This study focused on network analysis due to its proficiency in elucidating complex inter‐variable relationships. Future research employing these statistical approaches could enhance the comprehensiveness of temporal aspects and improve understanding of the trait resilience systems network. Consequently, these limitations indicate the need for further testing of the Dynamic Resilience Spectrum Theory to refine its aspects.

Future research can enhance these findings by implementing more sophisticated study designs and statistical analyses. This includes a detailed examination of various disturbances, extended study periods, and thorough investigation of both individual and contextual factors affecting resilience, with a focus on cultural differences. Longitudinal studies are vital for monitoring the development and consistency of trait resilience networks over time. This is particularly true in the context of disturbances, including systemic challenges like socioeconomic stress and immediate stressors like personal life events. This method will clarify the relationship between trait resilience networks and various disturbances, enhancing the understanding of resilience and various adversities.

This study reconceptualizes trait resilience as an ecological systems network, demonstrating network dynamics in United Kingdom and USA contexts. Study One highlights adaptive capacity as fundamental in a resilience systems network, linked with beneficial aspects of emotional processing. It proposes a Broaden‐and‐Build approach where adaptive capacity is a key component, occasionally in tandem with positive emotional processes. Conversely, Study Two's outcomes introduce the Dynamic Resilience Spectrum Theory. This theory posits that higher levels of disturbance drive a wider engagement with resilience traits compared to lower disturbance levels. Ultimately, the research advocates for a comprehensive, networked perspective in resilience studies, focusing on the inherent dynamism of individuals in utilizing diverse resilience traits.

## FUNDING INFORMATION

No funding was received for this research.

## CONFLICT OF INTEREST STATEMENT

No conflicts of interest exist for the research.

## ETHICS STATEMENT

This study received ethics approval from the corresponding author's Institutional Ethics Subject Board.

## Supporting information


Data S1.

## Data Availability

The study was preregistered, with an analysis plan, and data available at the Open Science Framework (doi: 10.17605/OSF.IO/3V48W). Two additions were made after the original preregistration. In Study One, preexisting data was incorporated (Samples 3 and 4) to replicate the findings. For Study Two, the Network Comparison Test was introduced to compare different network structures and the bootstrapped centrality difference test to assess node differences. These updates are documented in a revised version of the preregistration.
