# Supplementary material for: Networking trait resilience: Unifying fragmented trait resilience systems from an ecological systems theory perspective
Source: J Pers. 2024 Mar 1;93(2):216–32. doi: 10.1111/jopy.12925 (PMC11891989; doi:10.1111/jopy.12925)
Supplement: Supplementary file 1 — Data S1. [file JOPY-93-216-s001.docx]

Supplementary Material

Supplementary Material S1.

To assess the reliability of the new social cohesion measure, Cronbach’s alpha was used, which yielded a satisfactory value of α=.91 (Cronbach, 1951).

For convergent validity, the relationship with the new measure and the Positive Relationships with Others scale from the Mental Health Continuum Short Form (Keyes, 2009), which measures the quality of social connections were used. This new measure is inherently linked to social cohesion, as positive relationships with others are a core component of this, as they involve forming meaningful, supportive, and cooperative connections with others. Therefore, a measure of social cohesion is likely to correlate highly with the presence of positive relationships within a group or community. Additionally, the association of Social Cohesion with two personality dimensions from the Ten-Item Personality Inventory (TIPI) (Gosling et al., 2003): agreeableness, indicating harmonious interactions and supportive social environments, and extraversion, reflecting a tendency for social interaction and feeling energized in social settings, weas explored. However, as both are personal traits and not a direct measure of group or social dynamics, the correlation with social cohesion, while positive, may not be as high as with the measure of positive relationships.

For the zero-order correlations between the measures, the statistical significance and effect size using a frame of reference, with r ≥ .37 representing a large effect size, .24 ≤ r < .37 representing a moderate effect size, and .1 ≤ r < .24 representing a small effect size (Cohen et al., 2003; McGrath & Meyer, 2006), and a moderate effect size deemed to be the minimum at which the findings can be considered of practical significance (Cohen, 1992) were assessed. This criterion differs from the well-cited effect size of .1 = small to .5 = large, as Cohen based the comparisons with the d effect size criteria using a biserial correlation, while comparison with the d effect size for Pearson product moment correlation coefficients should be based on point biserial correlation (McGrath & Meyer, 2006). The findings revealed a large, significant positive correlation with 'positive relationships' (r = .46, p<.05), emphasizing its strong relevance to social cohesion. 'Agreeableness' showed a moderate, significant positive correlation (r = .276, p<.05), while 'extraversion' had a smaller but significant positive correlation (r = .219, p<.05). These results confirm the expected statistically significant relationships with social cohesion, each varying in magnitude, with the positive relationship for positive relationships with others being higher than the positive relationships with agreeableness and extraversion.

References

Cohen, J., Cohen, P., West, S. G., & Aiken, L. S. (2003). *Applied multiple regression/correlation analysis for the behavioral sciences* (3rd editio). Lawrence Erlbaum Associates Publishers.

Cronbach, L. J. (1951). Coefficient alpha and the internal structure of tests. *Psychometrika*, *16*(3), 297–334. https://doi.org/10.1007/BF02310555

Gosling, S. D., Rentfrow, P. J., & Swann, W. B. (2003). A very brief measure of the Big-Five personality domains. *Journal of Research in Personality*, *37*(6), 504–528. https://doi.org/10.1016/S0092-6566(03)00046-1

Keyes, C. (2009). Brief description of the mental health continuum short form (MHC-SF). *American Journal of Public Health*, *100*(12), 2366–2371.

McGrath, R. E., & Meyer, G. J. (2006). When effect sizes disagree: The case of r and d. *Psychological Methods*, *11*(4), 386–401. https://doi.org/10.1037/1082-989X.11.4.386

Supplementary Information S2.

*Principal Components Analysis loadings on the first component for the subscales used to create factor scores for Engineering, Ecological, Adaptive Capacity, and Social Cohesion Resilience, and Emotional Processing.*

| Engineering Resilience | | | |  | Ecological Resilience | | | |  | Emotional Processing | | | | |
| --- | --- | --- | --- | --- | --- | --- | --- | --- | --- | --- | --- | --- | --- | --- |
|  | Sample | | |  |  | Sample | | |  |  | Sample | | | |
|  | 1 | 3 | 4 |  |  | 1 | 3 | 4 |  |  | 1 | 2 | 3 | 4 |
| Brief Resilience | .93 | .94 | .94 |  | Planning Prioritizing Behaviour | .90 | .90 | .87 |  | Cognitive reappraisal | .55 | -.36 | .67 | .65 |
| Composure | .87 | .89 | .90 |  | Personal Competence | .80 | .81 | .82 |  | Expressive Suppression | -.62 | .77 | -.15 | -.20 |
| Engineering Resilience | .92 | .90 | .90 |  | Structured Style | .87 | .82 | .85 |  | PF Coping Engagement | .73 | -.60 | .56 | .60 |
|  |  |  |  |  |  |  |  |  |  | PF Coping Disengagement | -.33 | .56 | -.67 | -.65 |
| Adaptive Capacity Resilience | | | |  | Social Cohesion Resilience | | | |  | EF Coping Engagement | .73 | -.77 | .63 | .68 |
|  | Sample | | |  |  | Sample | | |  | EF Coping Disengagement | -.71 | .72 | -.61 | -.68 |
|  | 1 | 3 | 4 |  |  | 1 | 3 | 4 |  |  |  |  |  |  |
| Social Competence | .91 | .90 | .91 |  | Psychological Care | .91 | .93 | .93 |  |  |  |  |  |  |
| Adaptive-Capacity | .66 | .71 | .76 |  | Family Cohesion | .91 | .92 | .92 |  |  |  |  |  |  |
| Social Skills | .91 | .90 | .93 |  | Social Support | .89 | .93 | .91 |  |  |  |  |  |  |

Key: PF = Problem Focussed; EF = Emotion Focussed. Note factor scores for Emotional Processing for Sample 2 are that lower levels reflect higher levels of emotional processing.

| 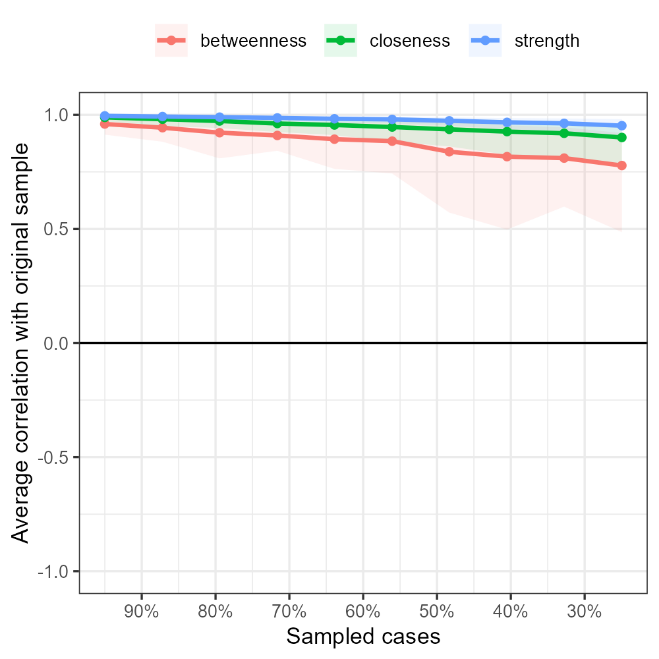 | 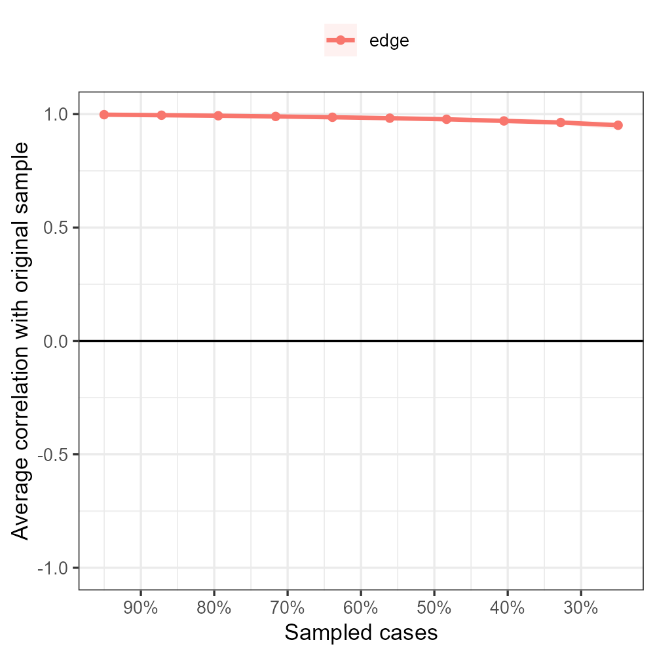 |
| --- | --- |
| USA; *n* = 1202 (Sample 1) | |
| 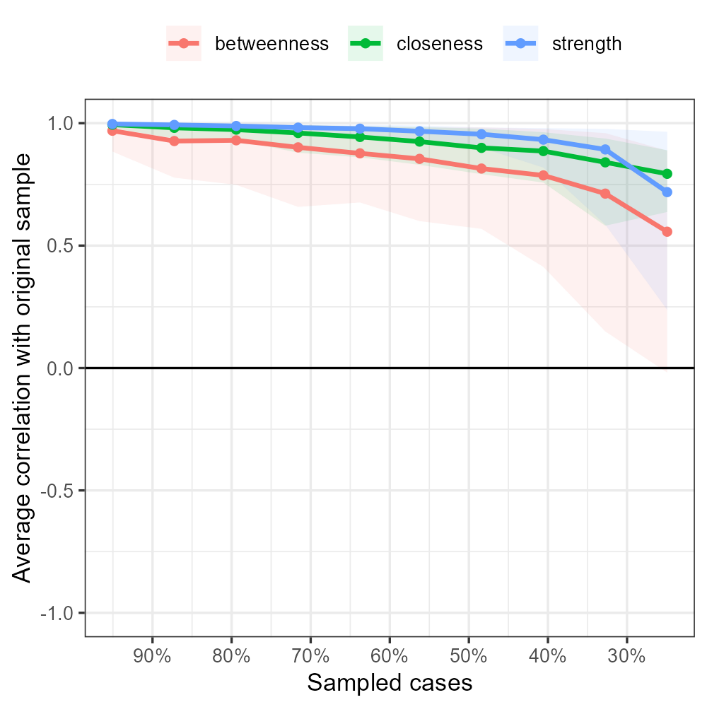 | 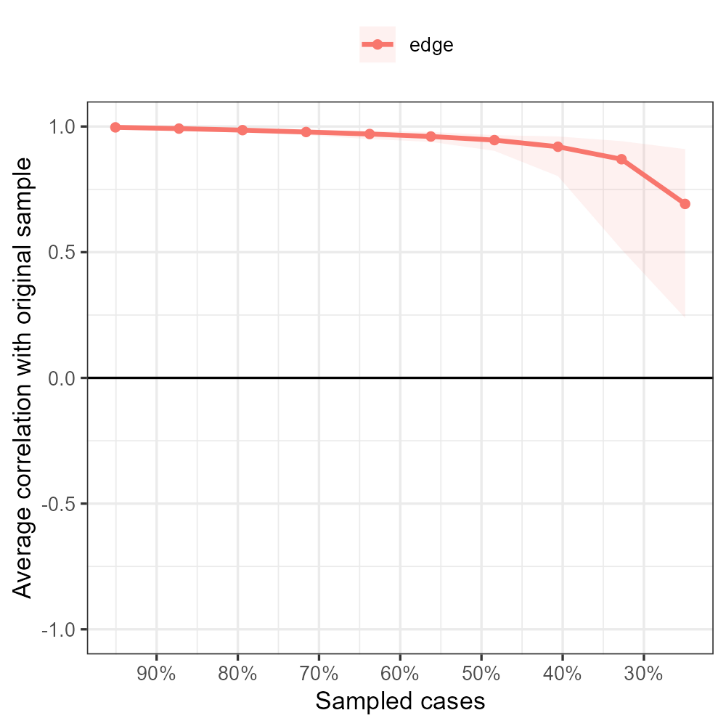 |
| UK; *n* = 345 (Sample 2) | |
| 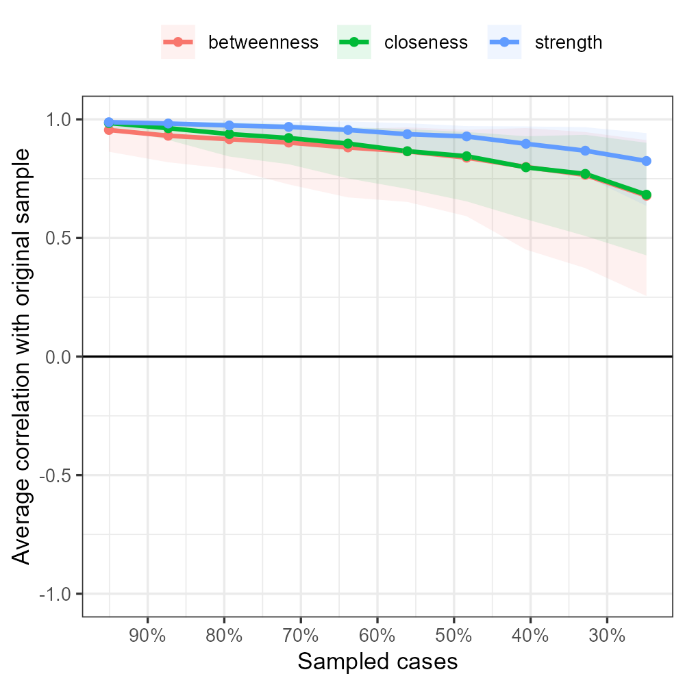 | 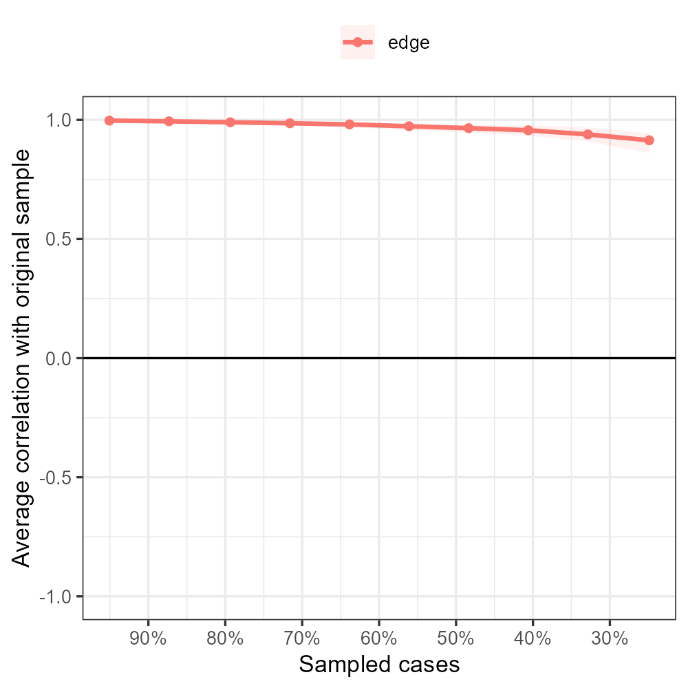 |
| USA; *n* = 426 (Sample 3) | |
| 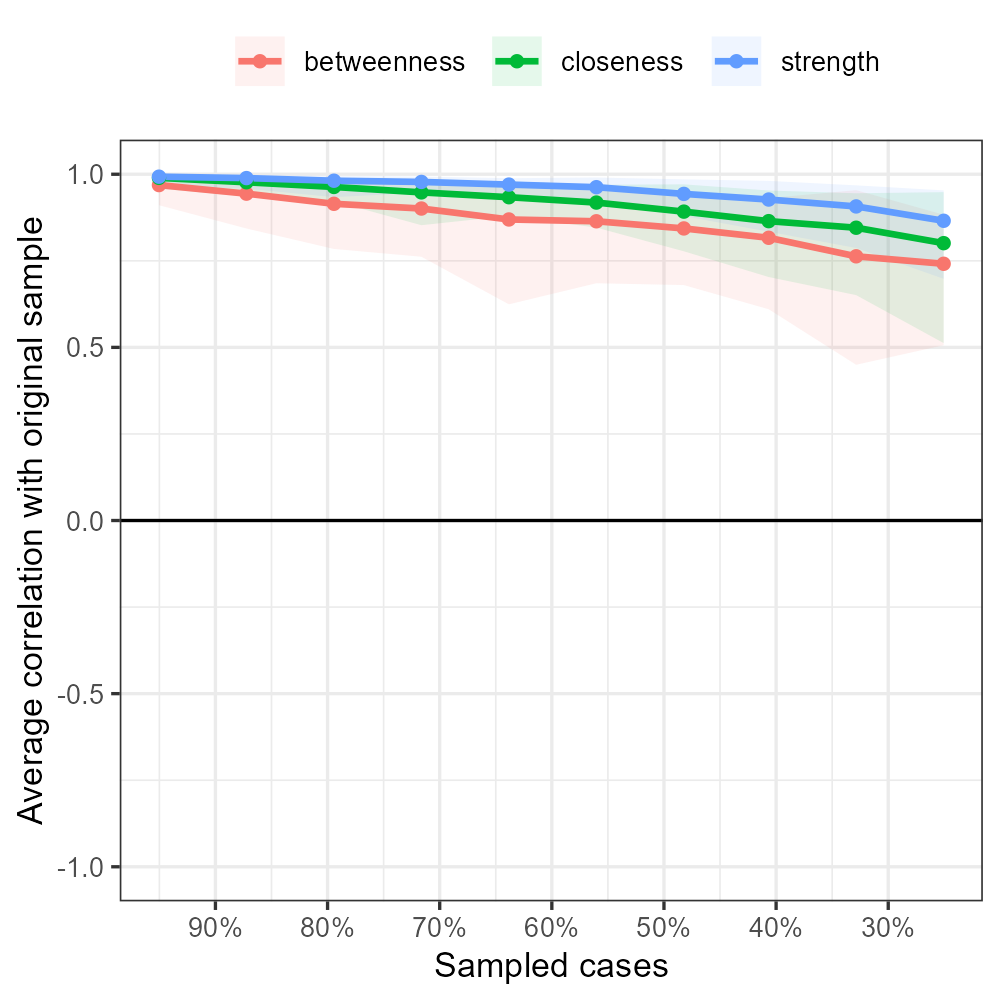 | 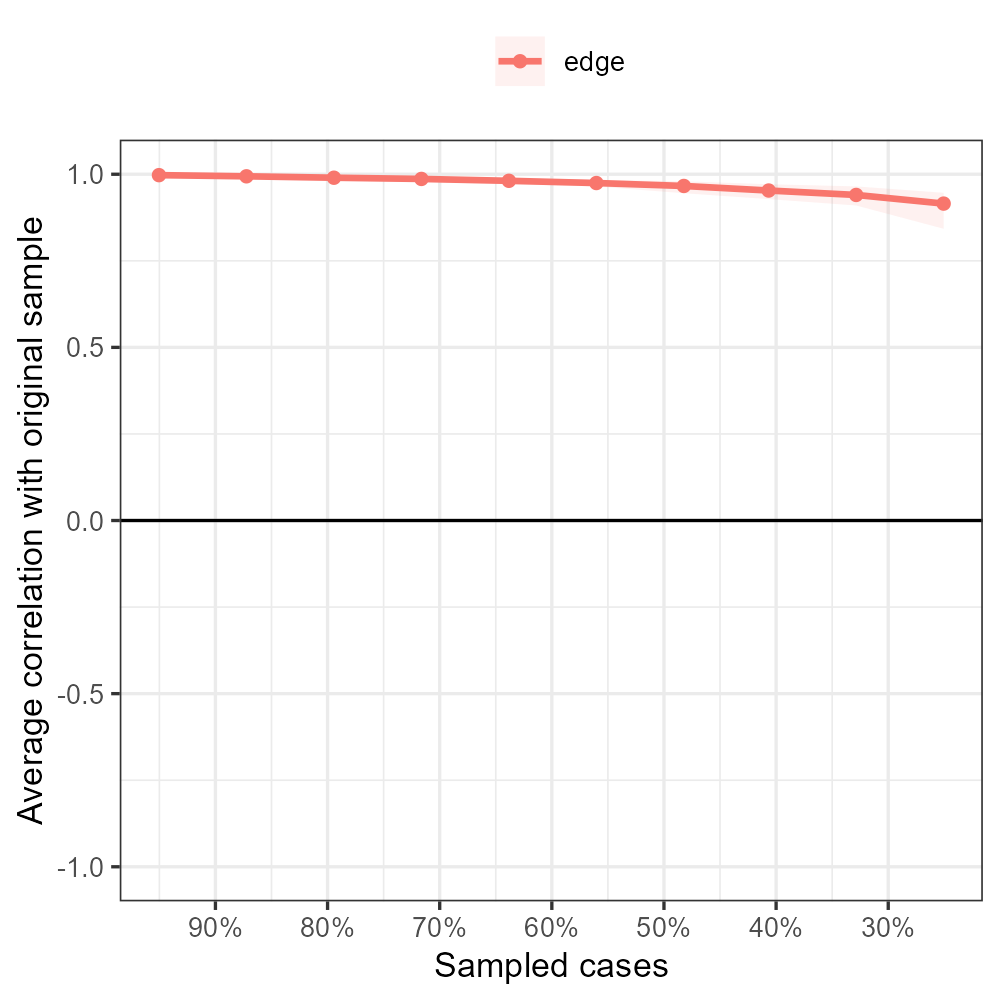 |
| UK; *n* = 423 (Sample 4) | |

Supplementary Material S3

*Edge and Centrality Stability Statistics for each trait resilience systems network by sample.*

| 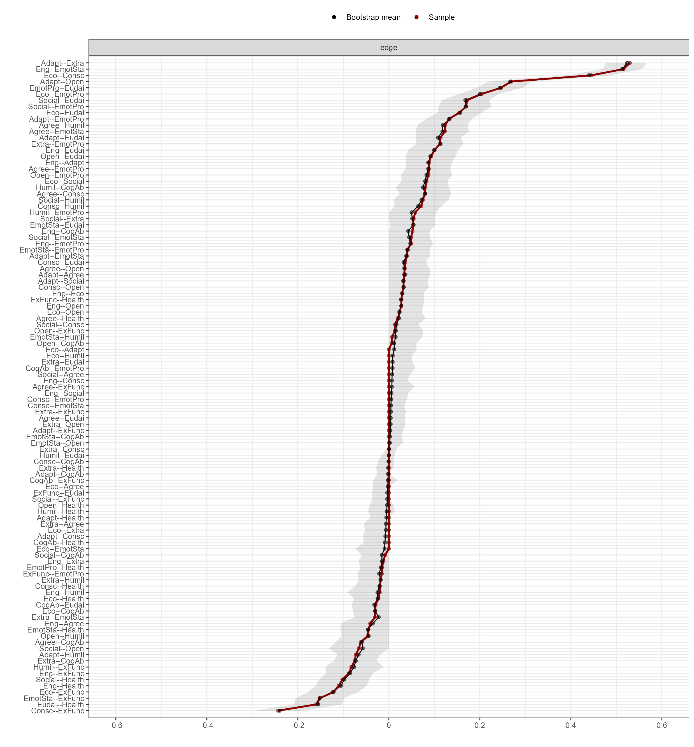 | 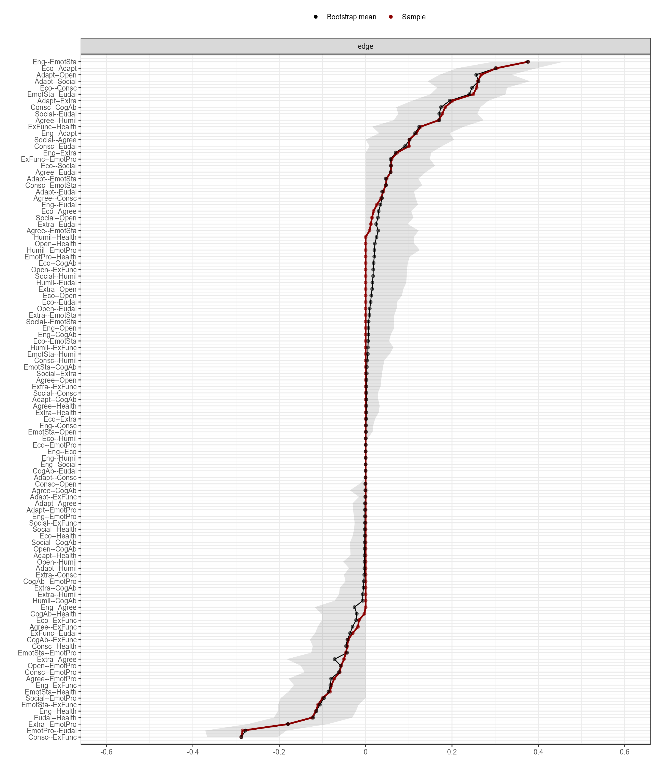 |
| --- | --- |
| USA; *n* = 1202 (Sample 1) | UK; *n* = 345 (Sample 2) |
| 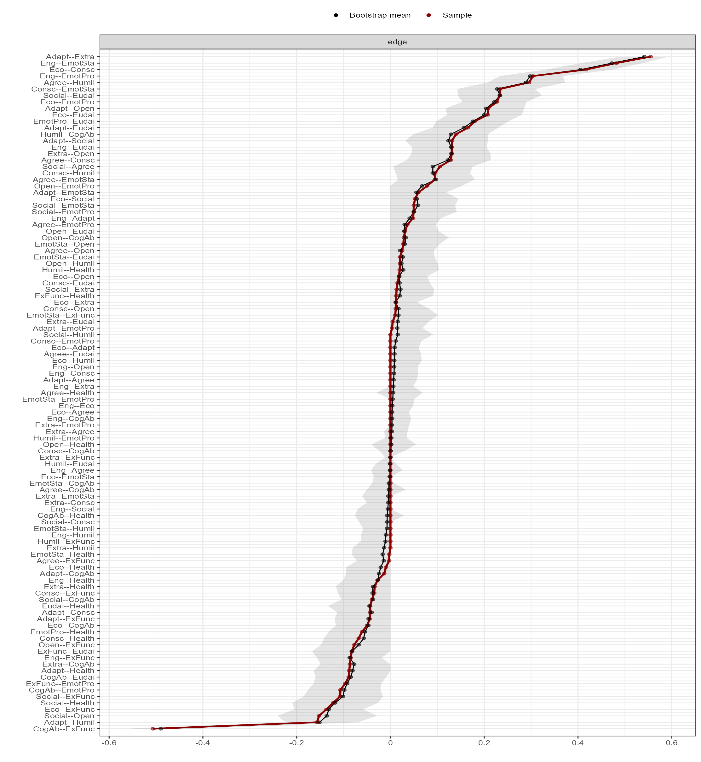 | 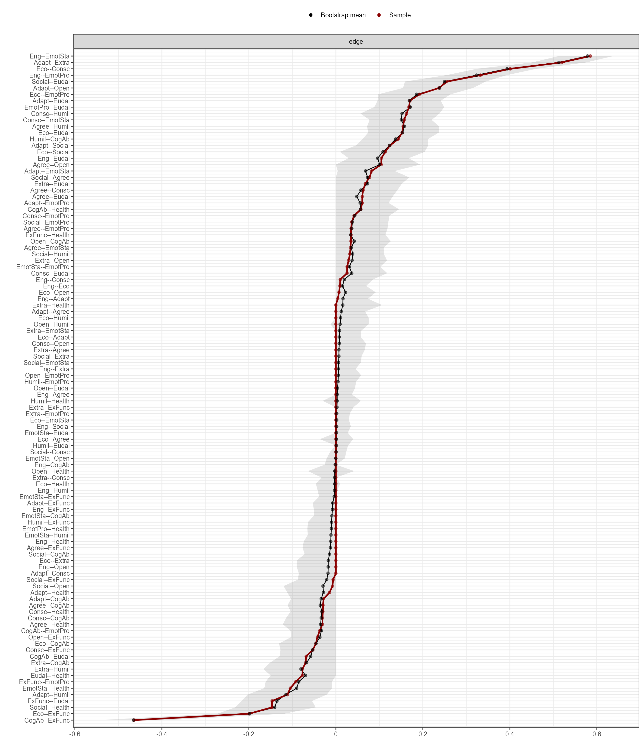 |
| USA; *n* = 426 (Sample 3) | UK; *n* = 423 (Sample 4) |

Note*:* Black line represents bootstrap mean, red line represents point-estimates of each edge weight and the grey shading shows the edge weight 95% confidence intervals. Wide intervals indicate lower stability and narrower intervals indicate higher stability.

Supplementary Material S4

*Bootstrapped confidence intervals of edge weights for every pairwise node comparison of the resilient systems network by Sample*

| 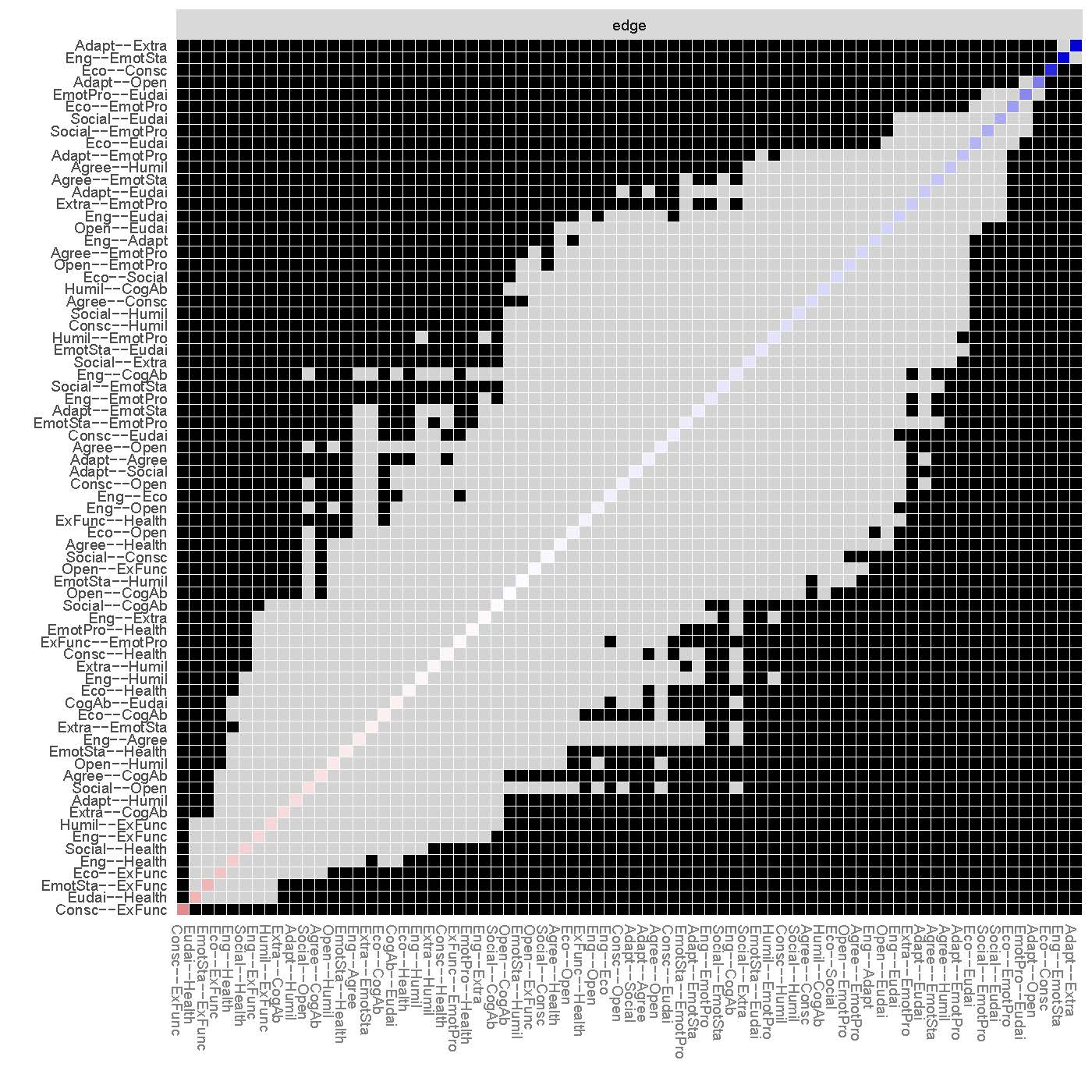 | 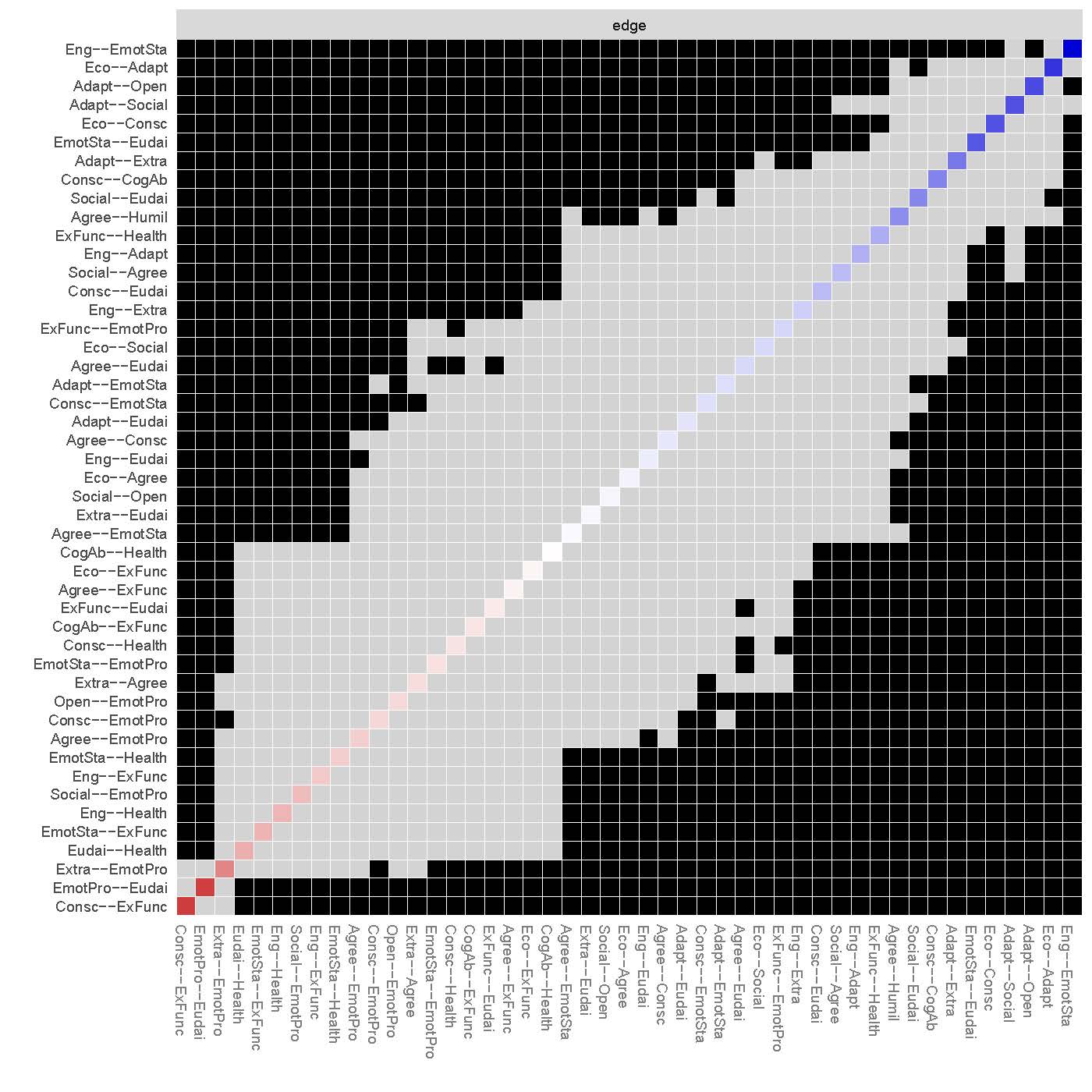 |
| --- | --- |
| USA; *n* = 1202 (Sample 1) | UK; *n* = 345 (Sample 2) |
| 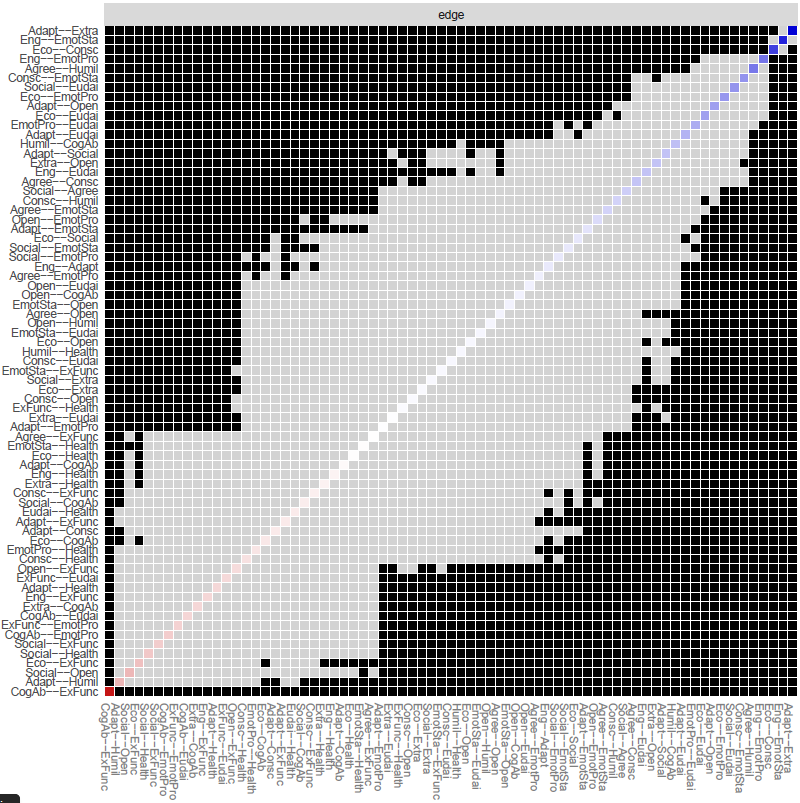 | 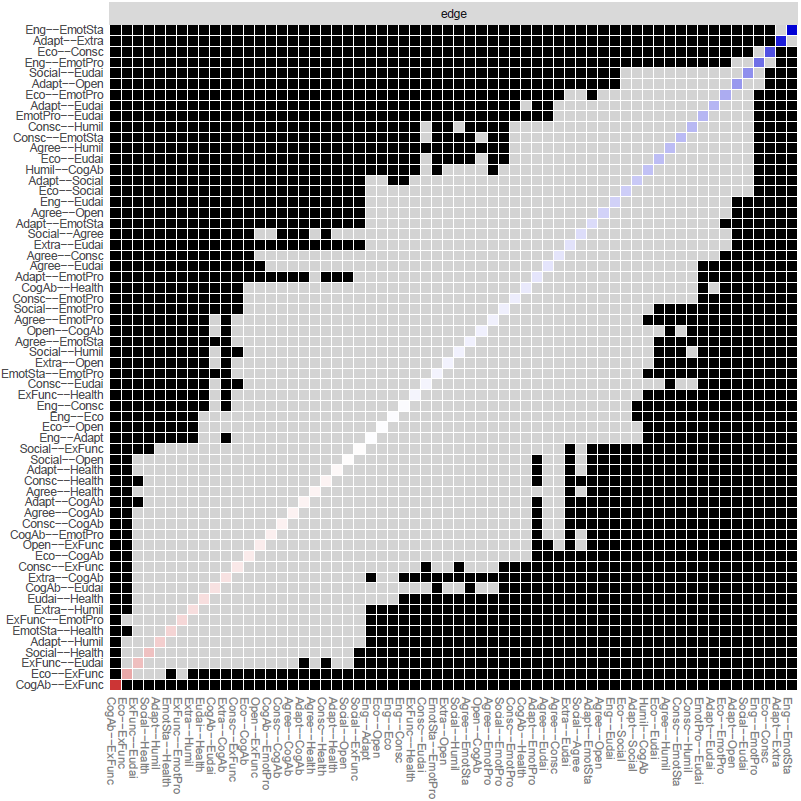 |
| USA; *n* = 426 (Sample 3) | UK; *n* = 423 (Sample 4) |

Note: Blue boxes correspond to positive correlations, while red boxes indicate negative correlations. Grey boxes indicate that the edge do not significantly differ (*p*>.05). Black boxes represent significant difference between the edges (*p*<.05).

Supplementary Material S5

*Bootstrapped edge weights difference test for the trait resilience systems network by sample.*

| 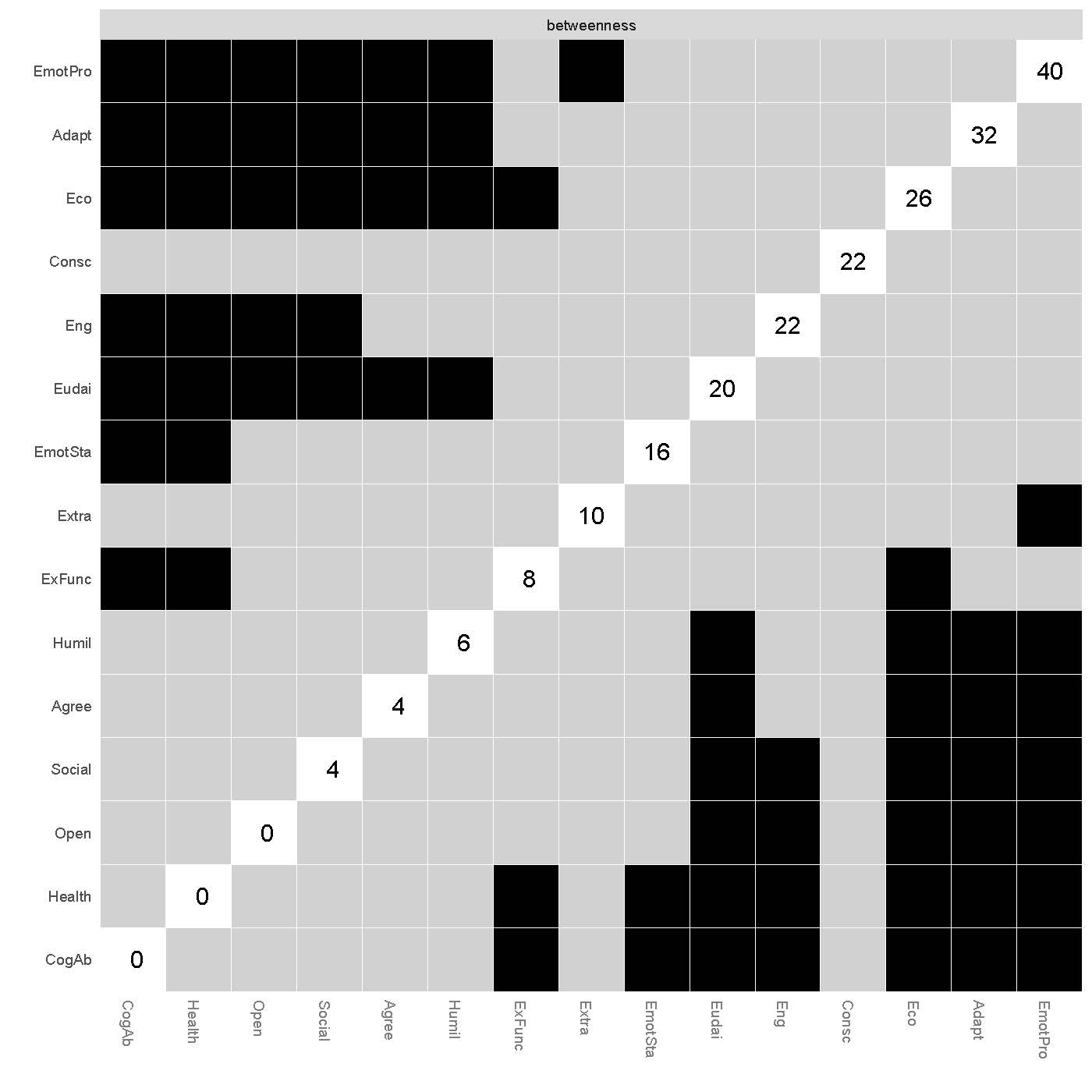 | 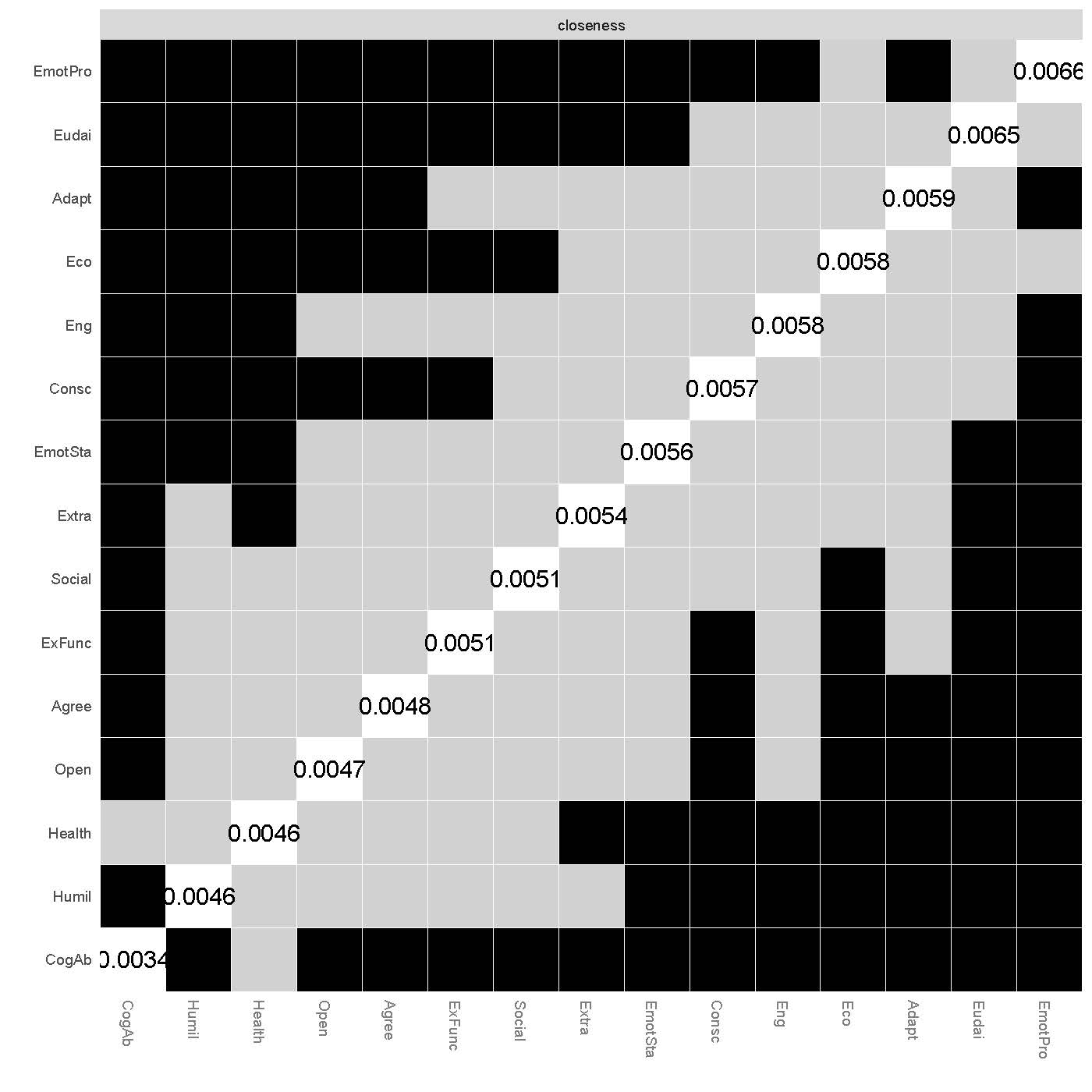 |
| --- | --- |
| USA; *n* = 1202 (Sample 1) | |
| 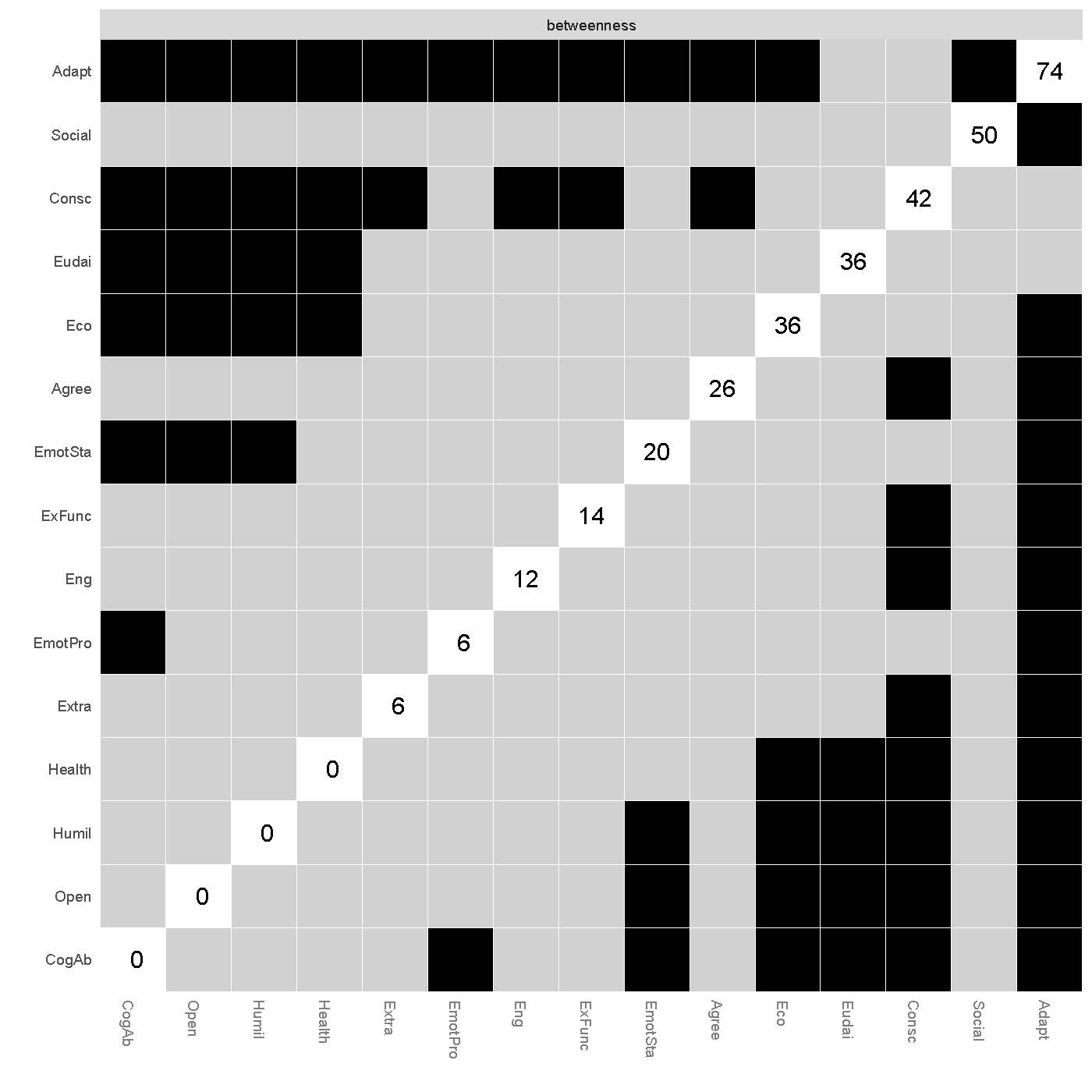 | 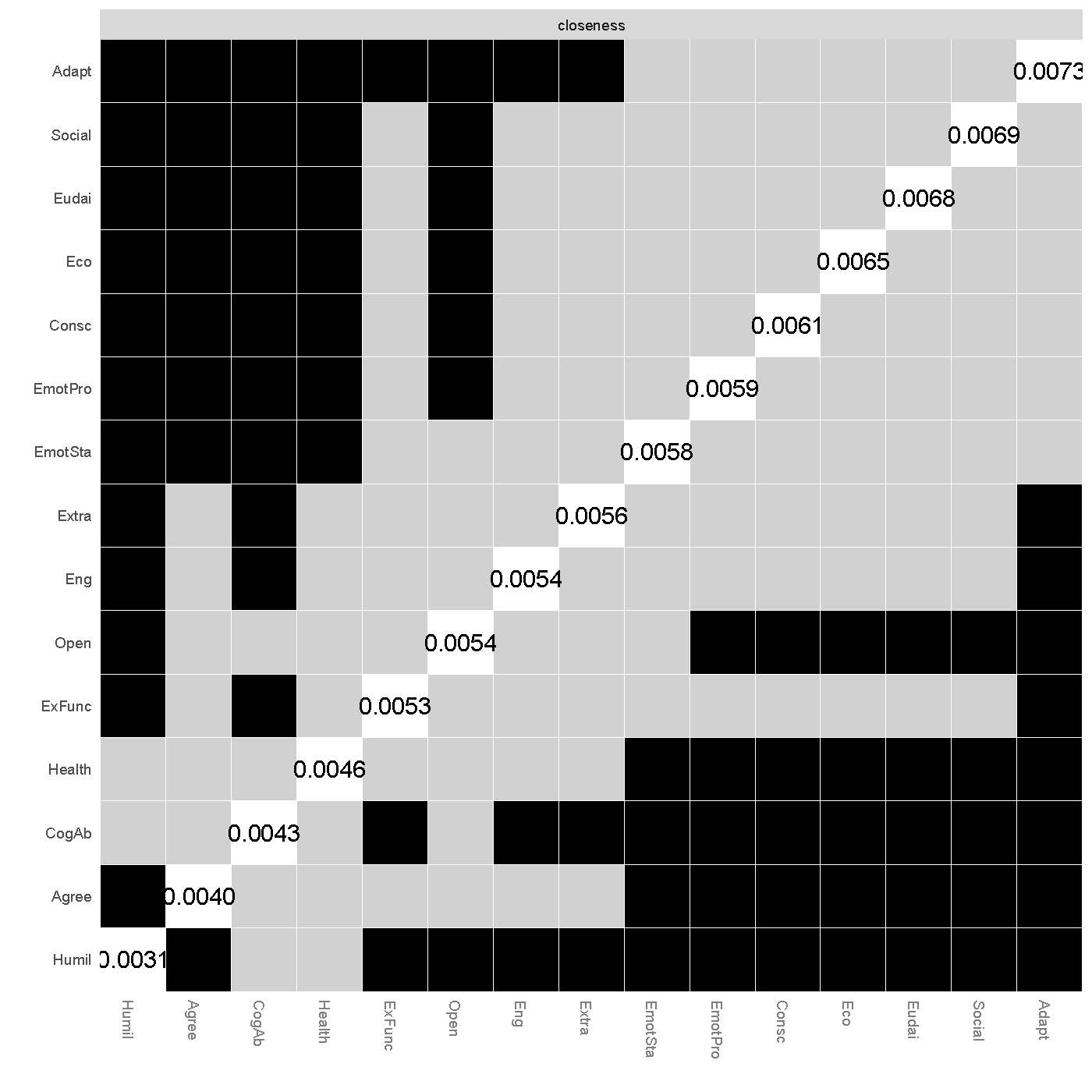 |
| UK; *n* = 345 (Sample 2) | |
| 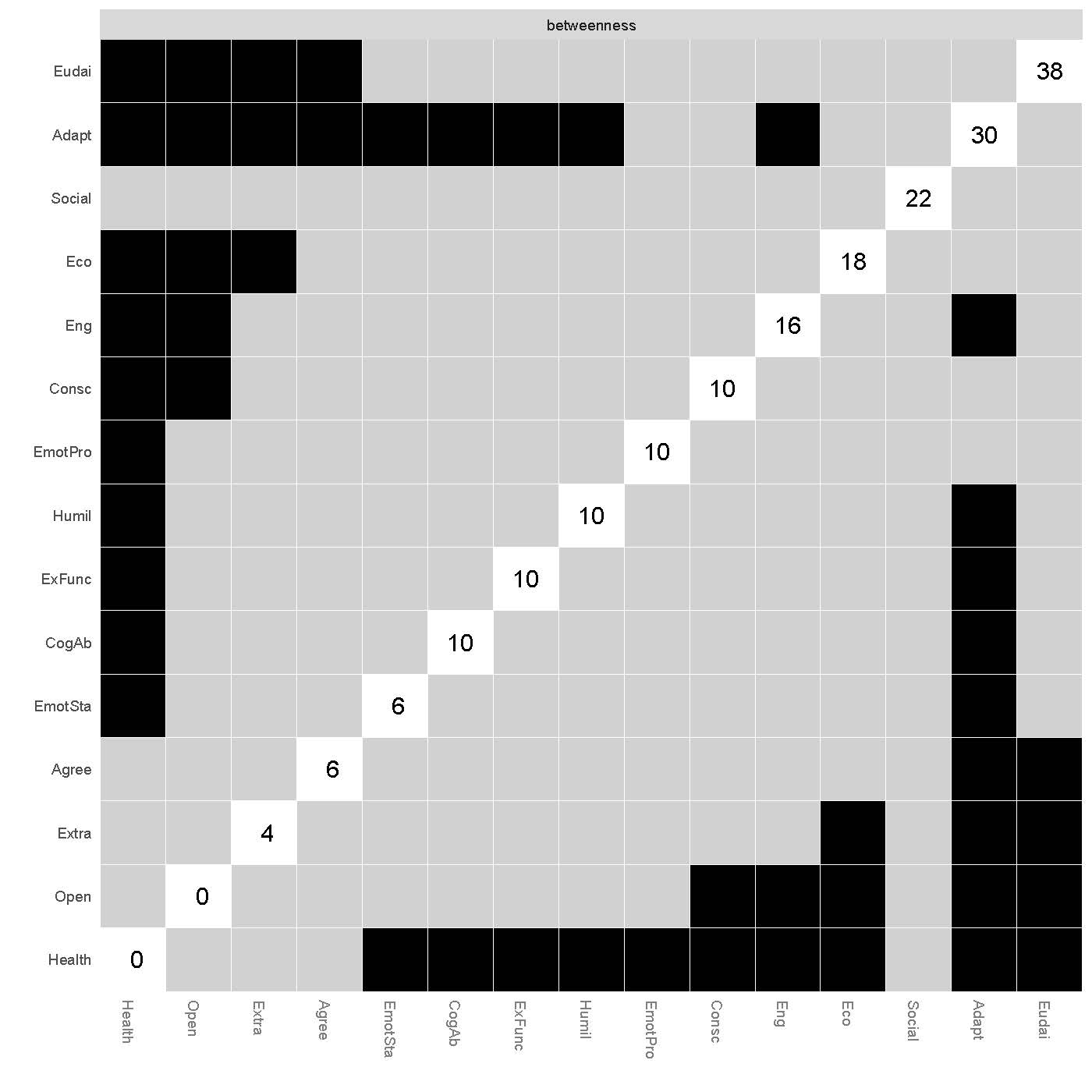 | 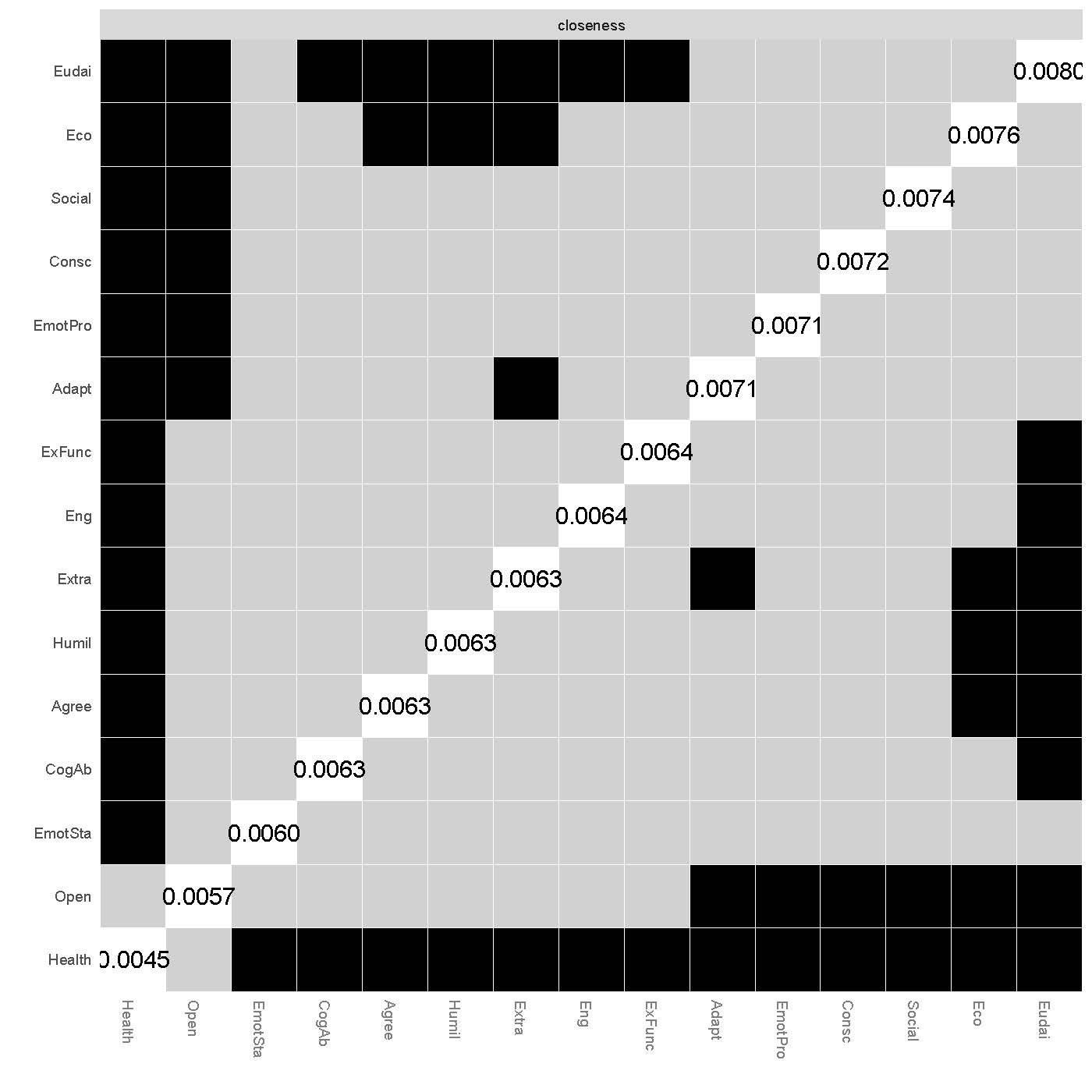 |
| USA; *n* = 426 (Sample 3) | |
| 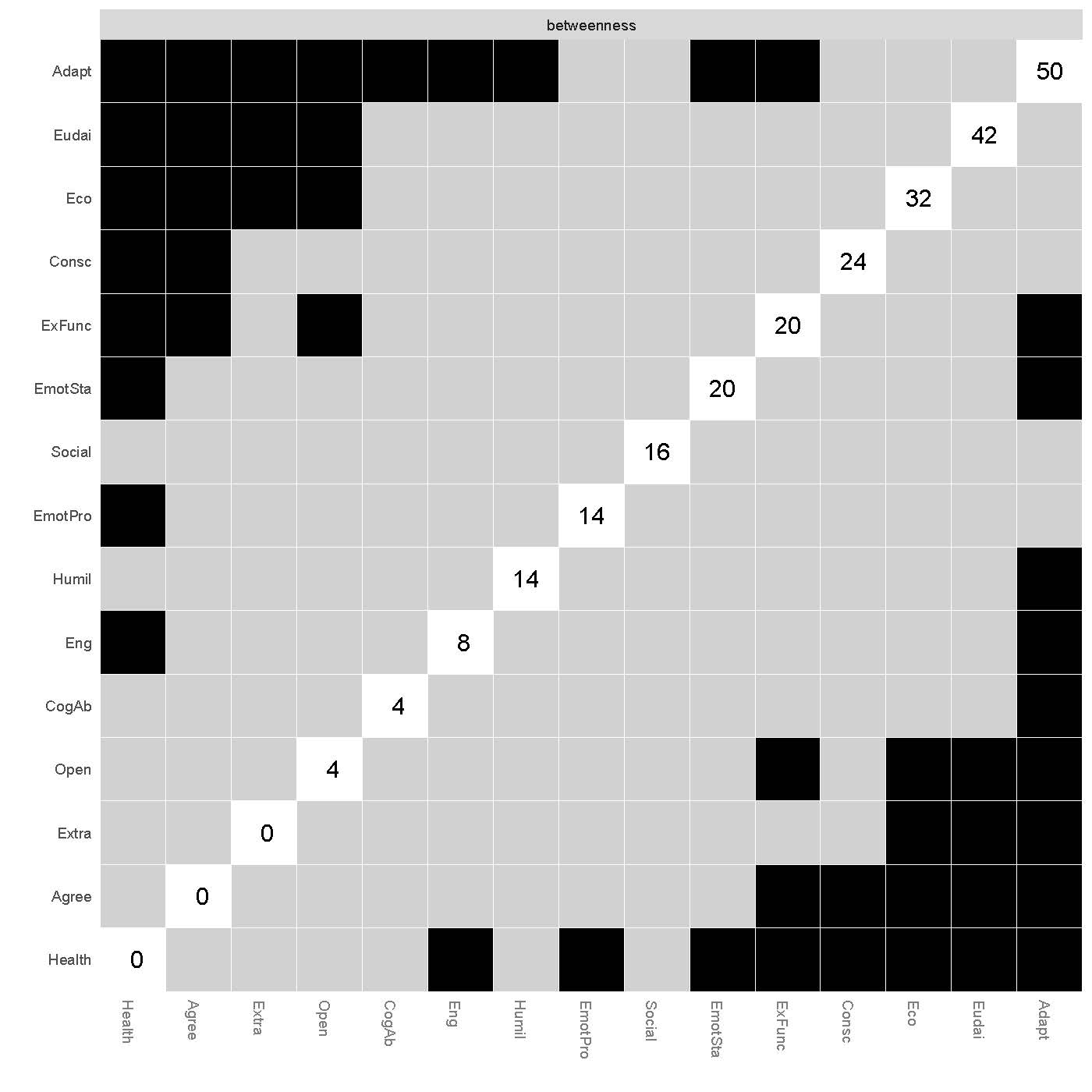 | 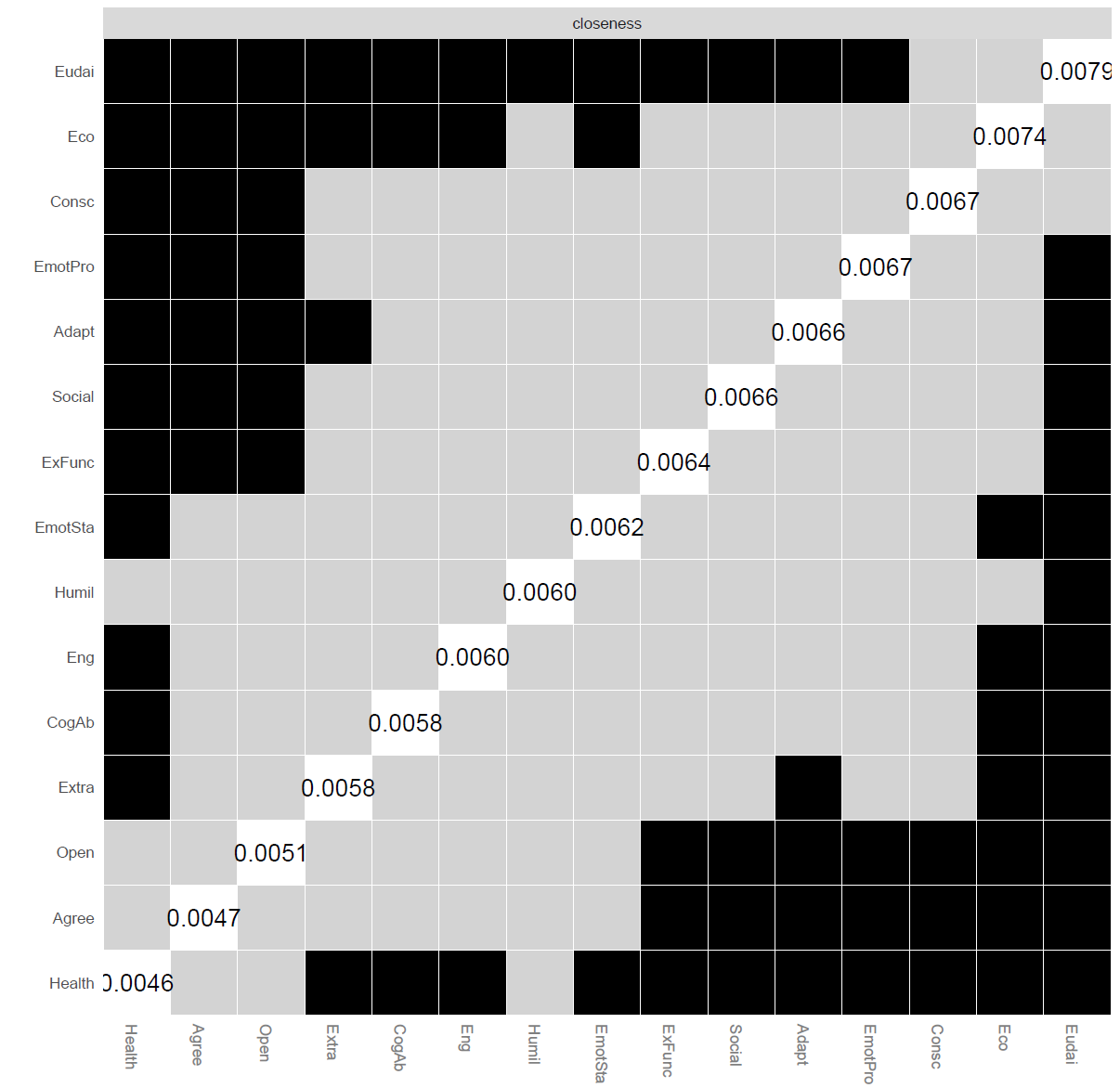 |
| UK; *n* = 423 (Sample 4) | |

*Note*: This test presents the bootstrapped significance of betweenness and closeness centrality estimates for each pairwise node comparison for the trait resilience systems network White boxes represent the values of node strength. Gray boxes indicate that the nodes do not significantly differ (*p*>.05). Black boxes represent significant difference (*p*<.05).

Supplementary Material S6

*Bootstrapped centrality difference test for betweenness and closeness nodes for each sample*

| 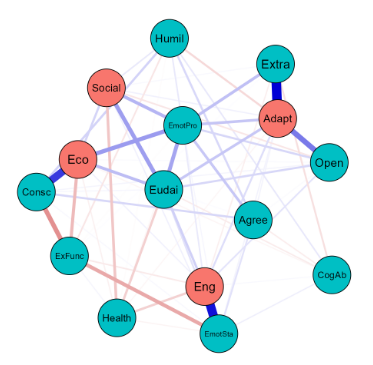 | 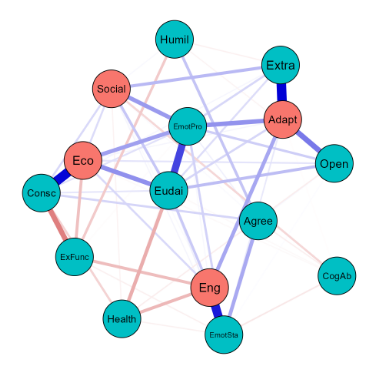 |
| --- | --- |
| Time 1 (Lower Disturbance) | Time 1 (Higher Disturbance) |
| 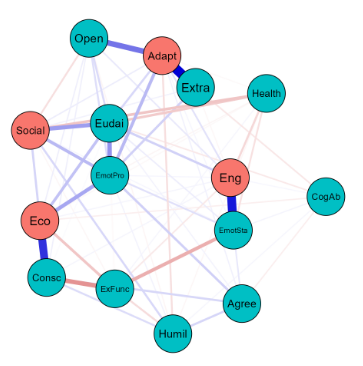 | 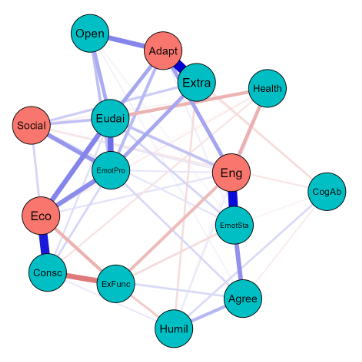 |
| Time 2 (Lower Disturbance) | Time 2 (Higher Disturbance) |
| *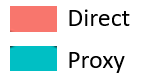* | |

Supplementary Material S7

*Illustrations of the trait resilience network systems among four samples for lower and higher disturbance at Time 1 and Time 2*

| 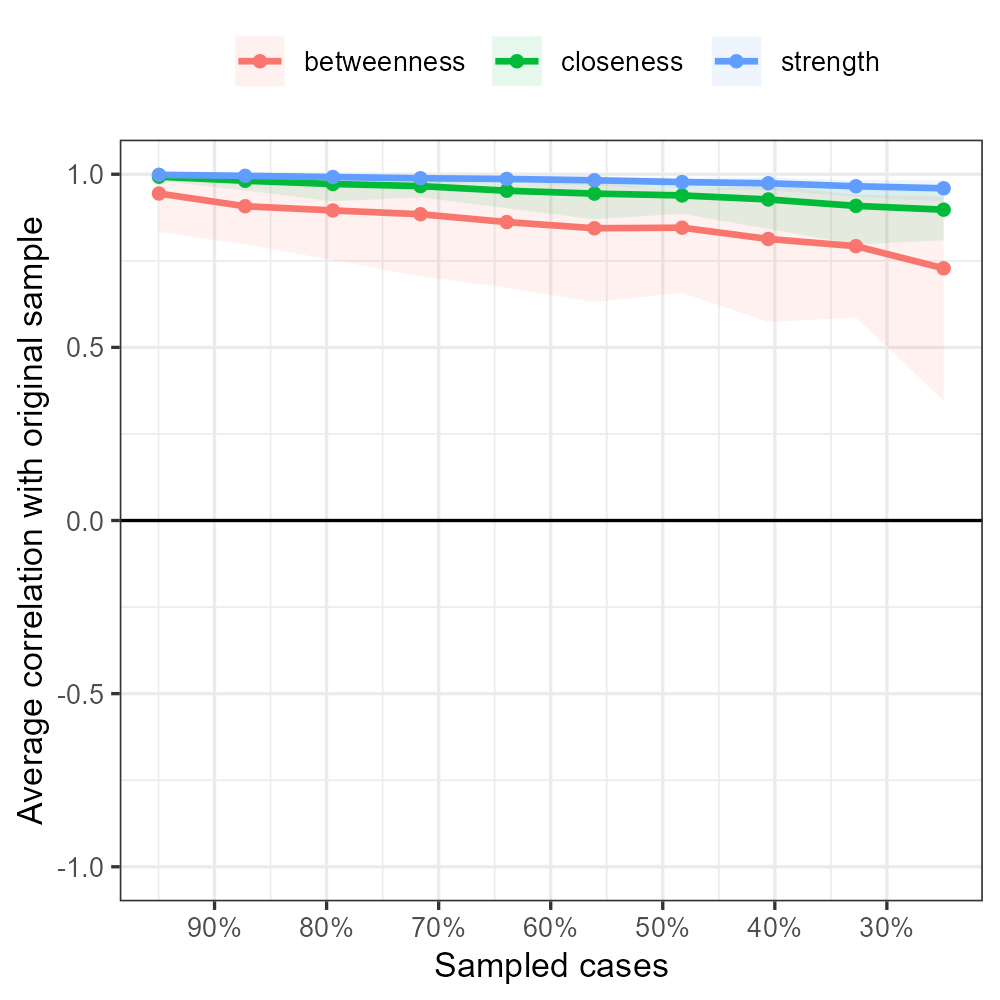 | 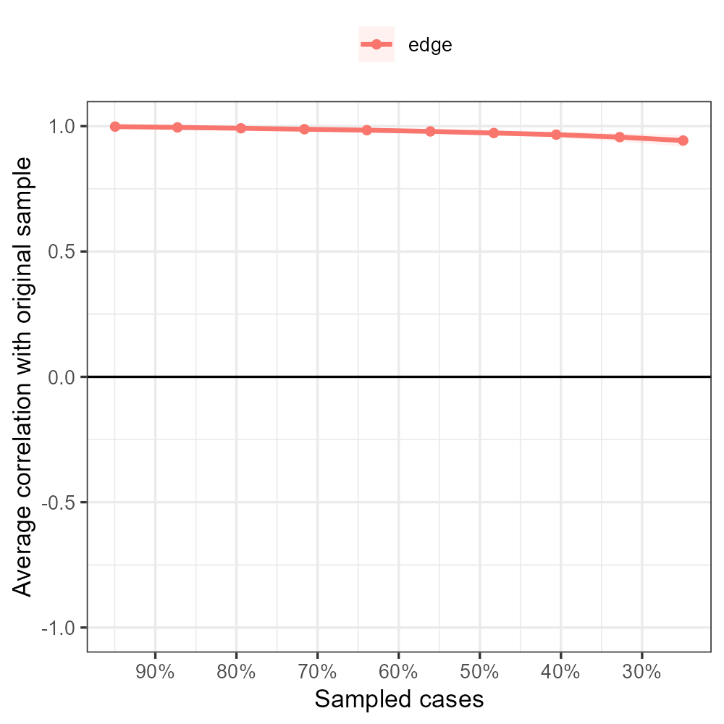 |
| --- | --- |
| Time 1 (Lower Disturbance) | |
| 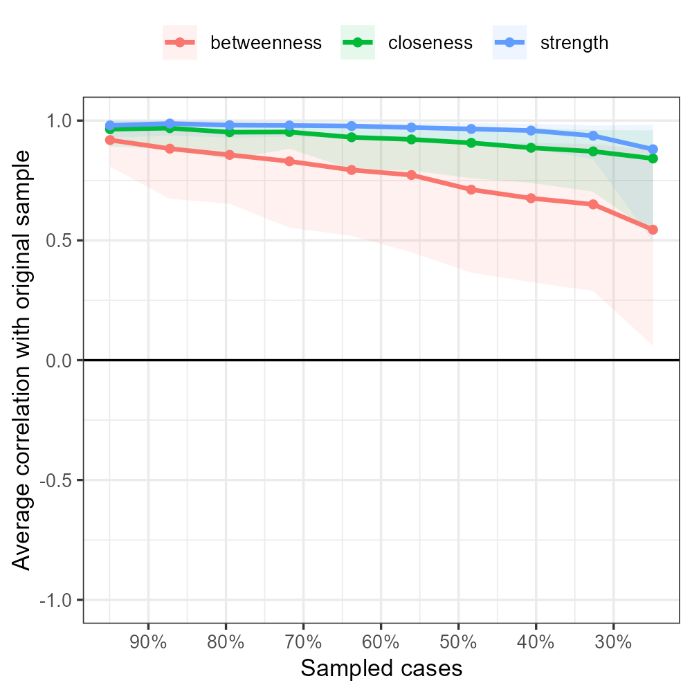 | 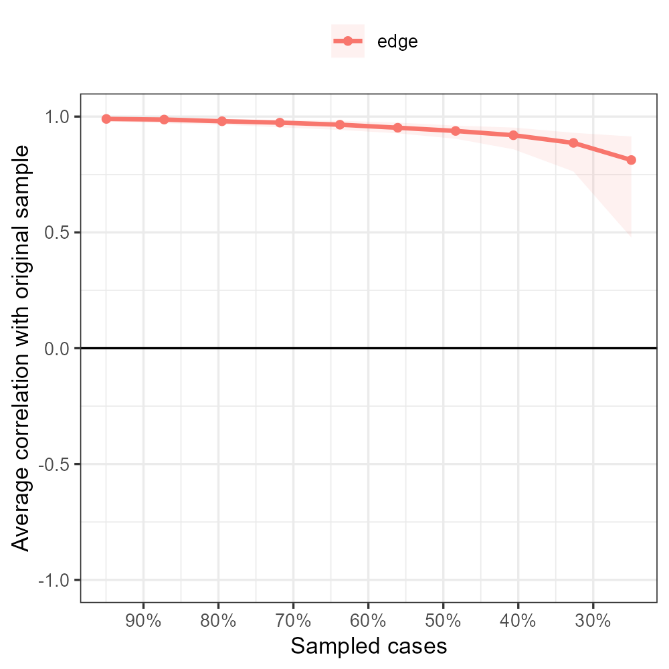 |
| Time 1 (Higher Disturbance) | |
| 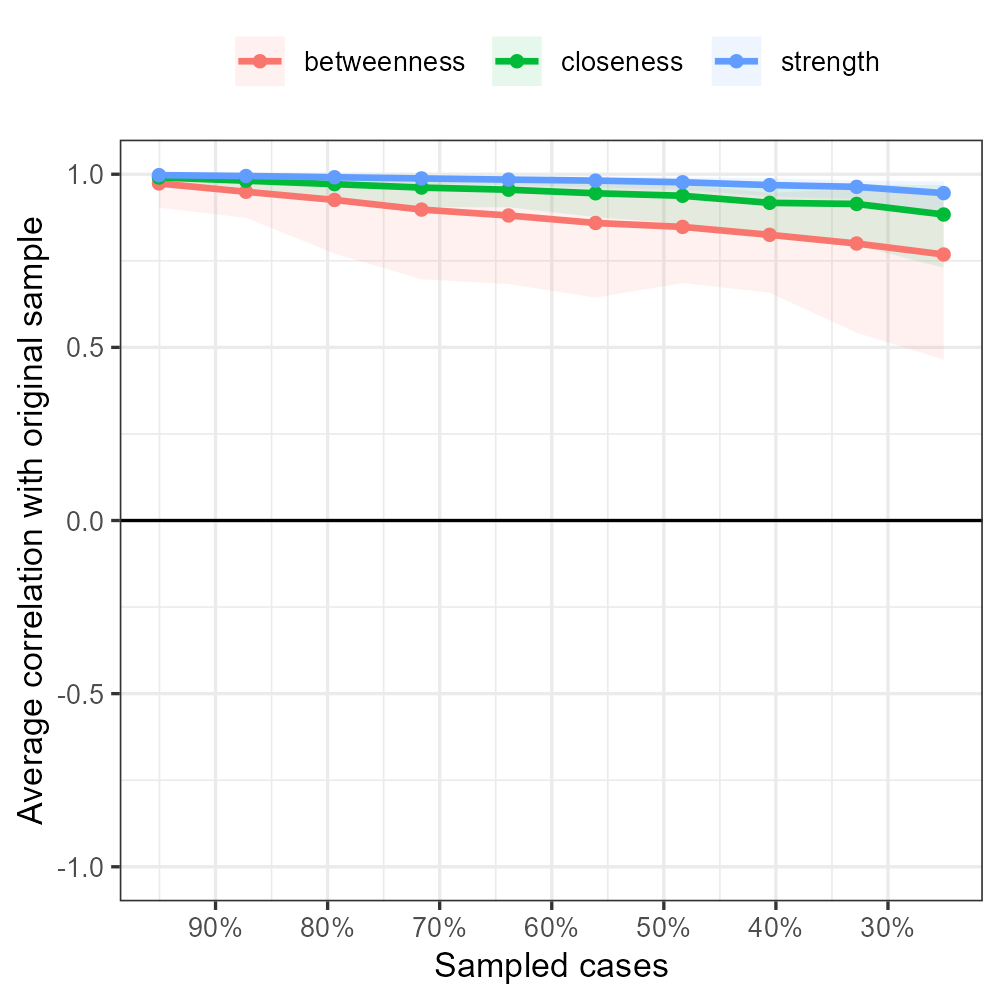 | 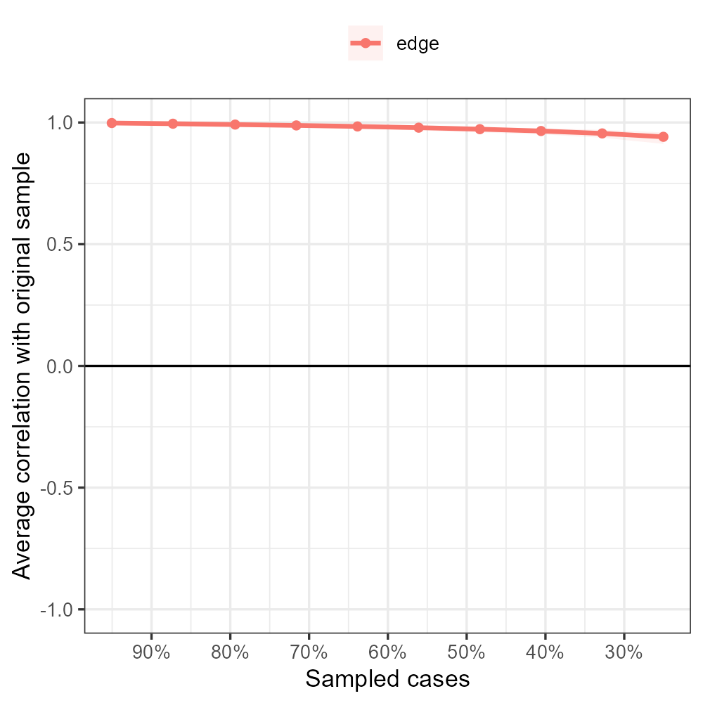 |
| Time 2 (Lower Disturbance) | |
| 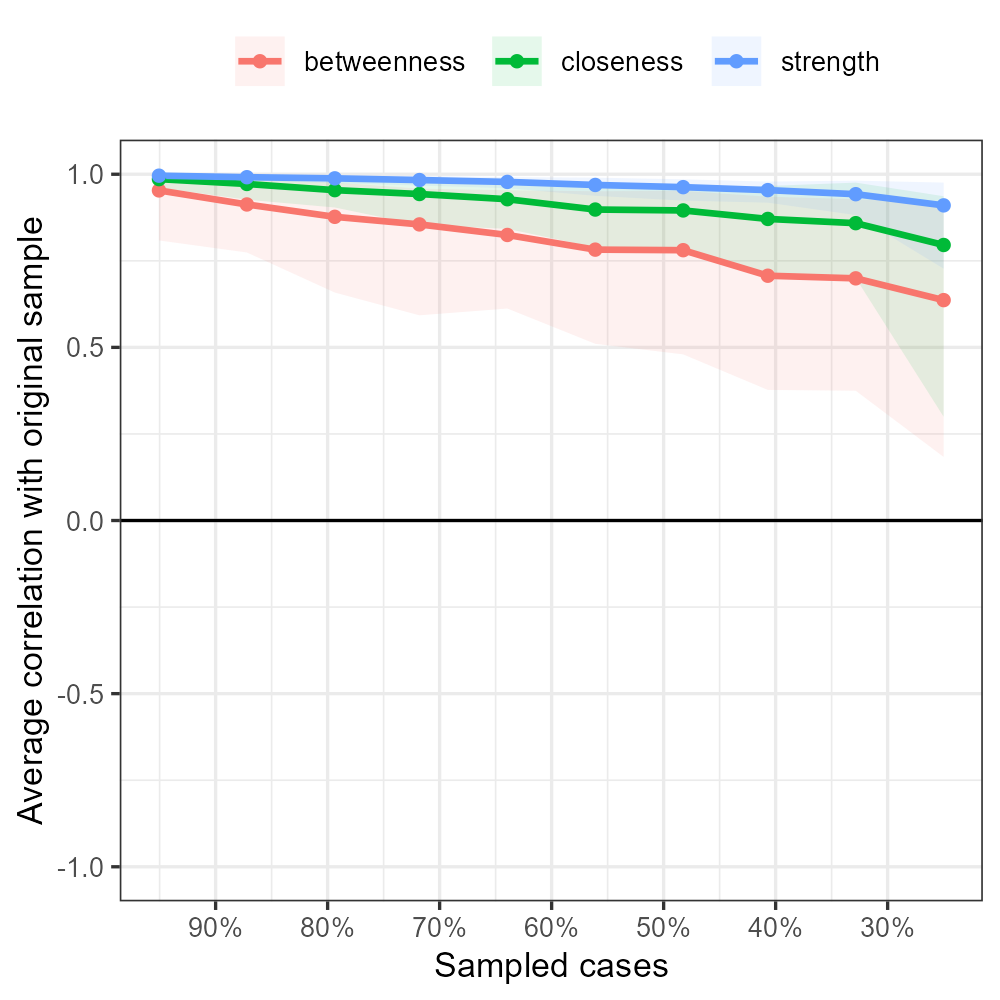 | 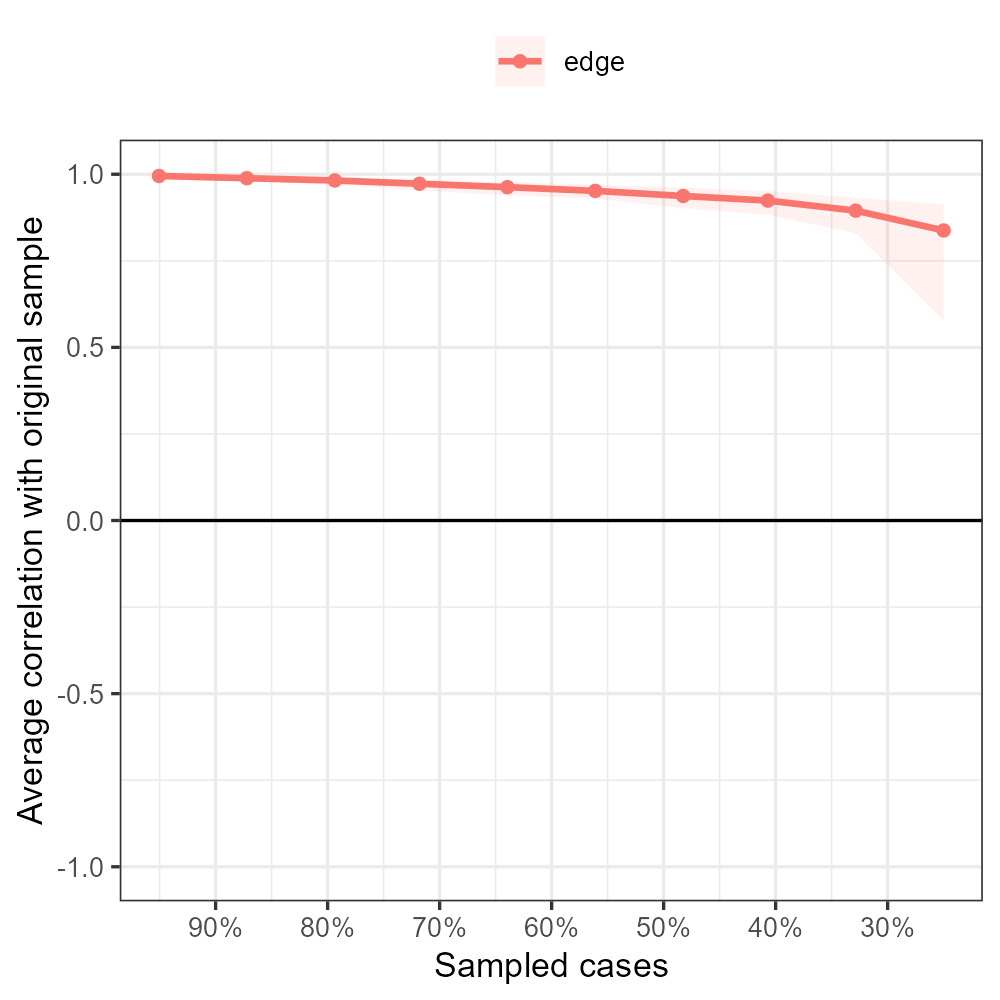 |
| Time 2 (Higher Disturbance) | |

Supplementary Material S8

*Edge and Centrality Stability Statistics for each trait resilience systems network by Time and Disturbance status*

| 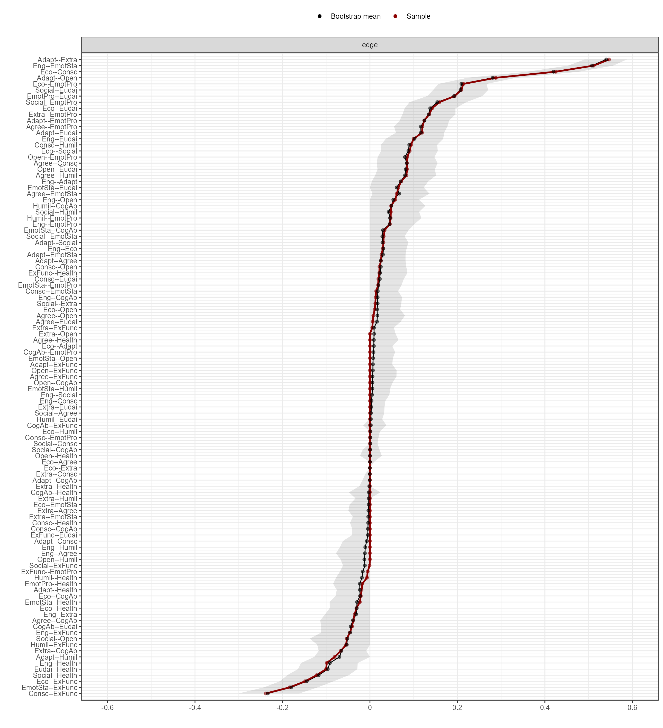 | 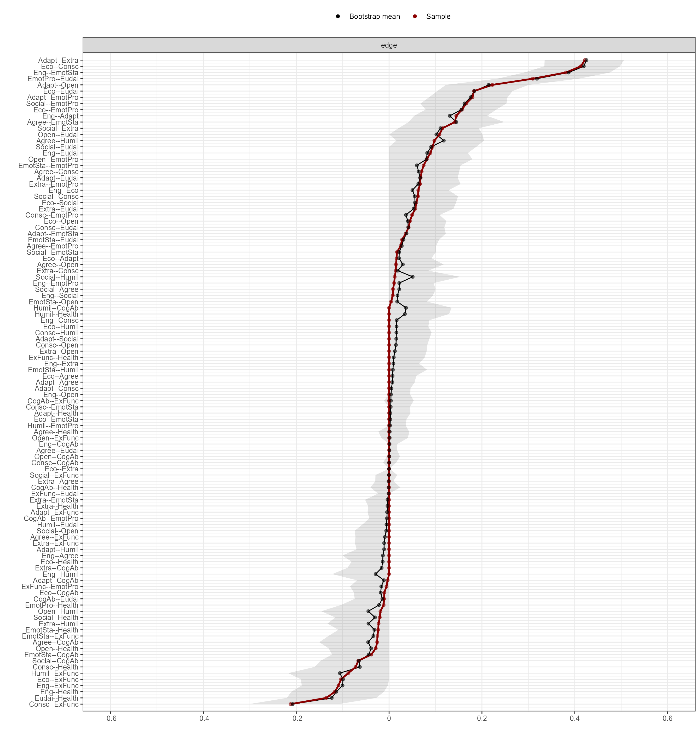 |
| --- | --- |
| Time 1 (Lower Disturbance) | Time 1 (Higher Disturbance) |
| 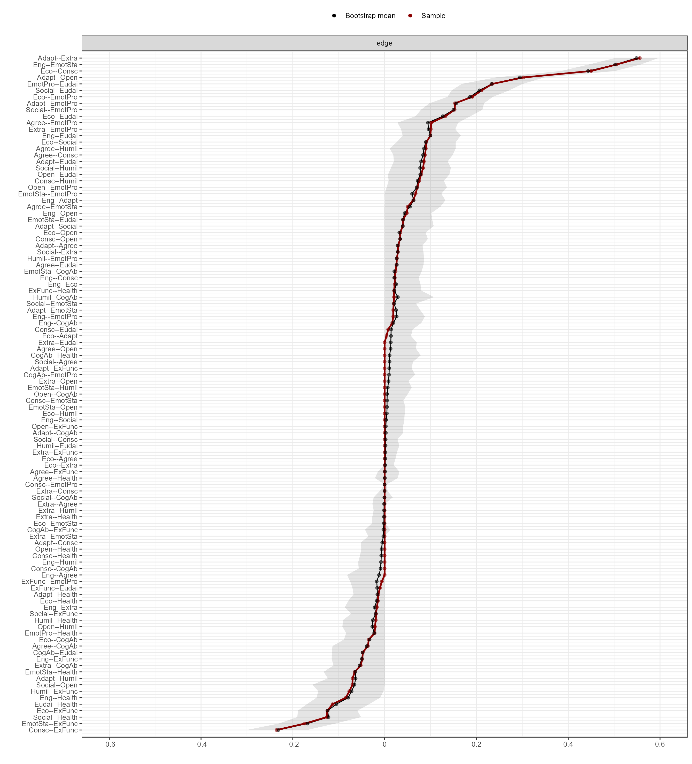 | 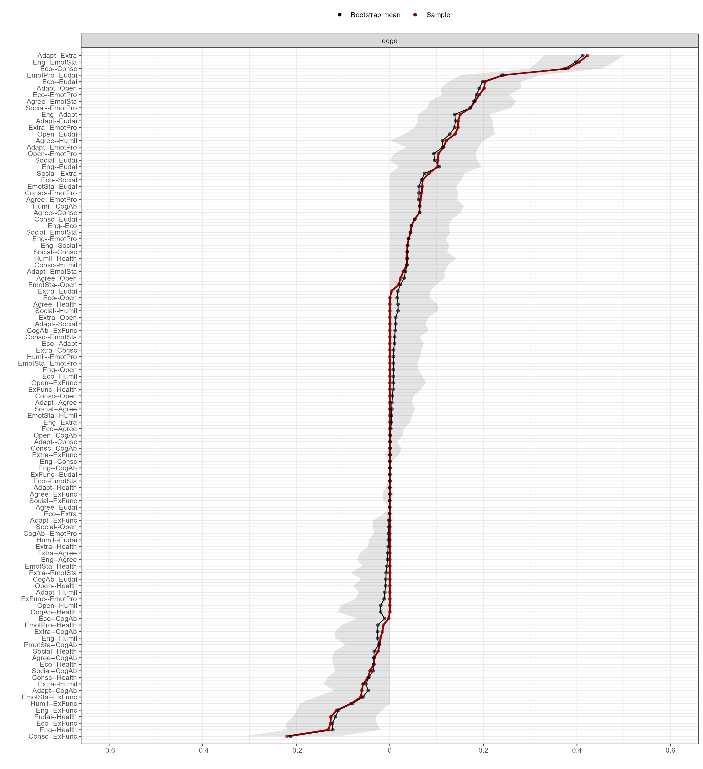 |
| Time 2 (Lower Disturbance) | Time 2 (Higher Disturbance) |

Note*:* Black line represents bootstrap mean, red line represents point-estimates of each edge weight and the grey shading shows the edge weight 95% confidence intervals. Wide intervals indicate lower stability and narrower intervals indicate higher stability.

Supplementary Material S9

*Bootstrapped edge weights for* *every pairwise node comparison of the resilient systems network by Time and Disturbance status.*

| 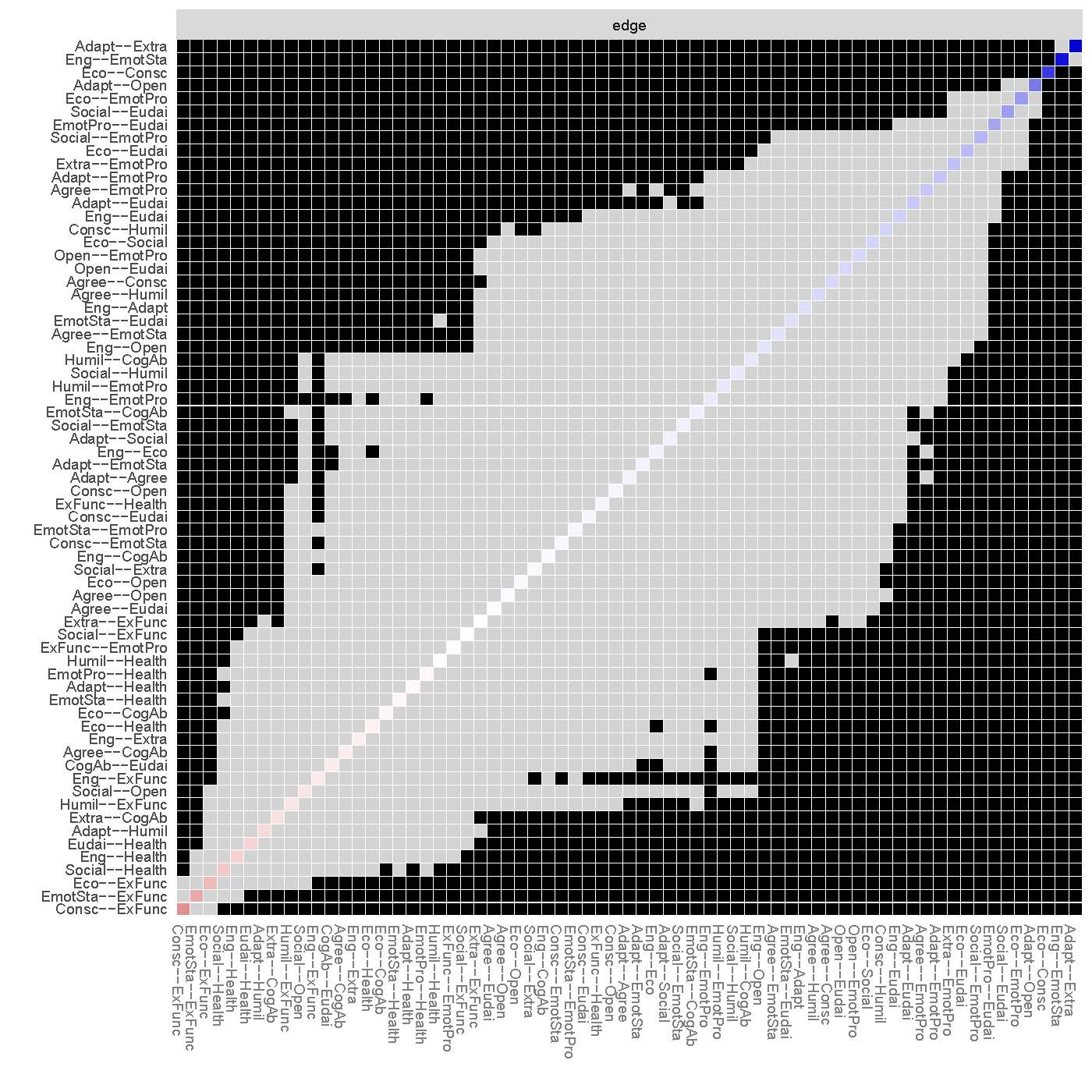 | 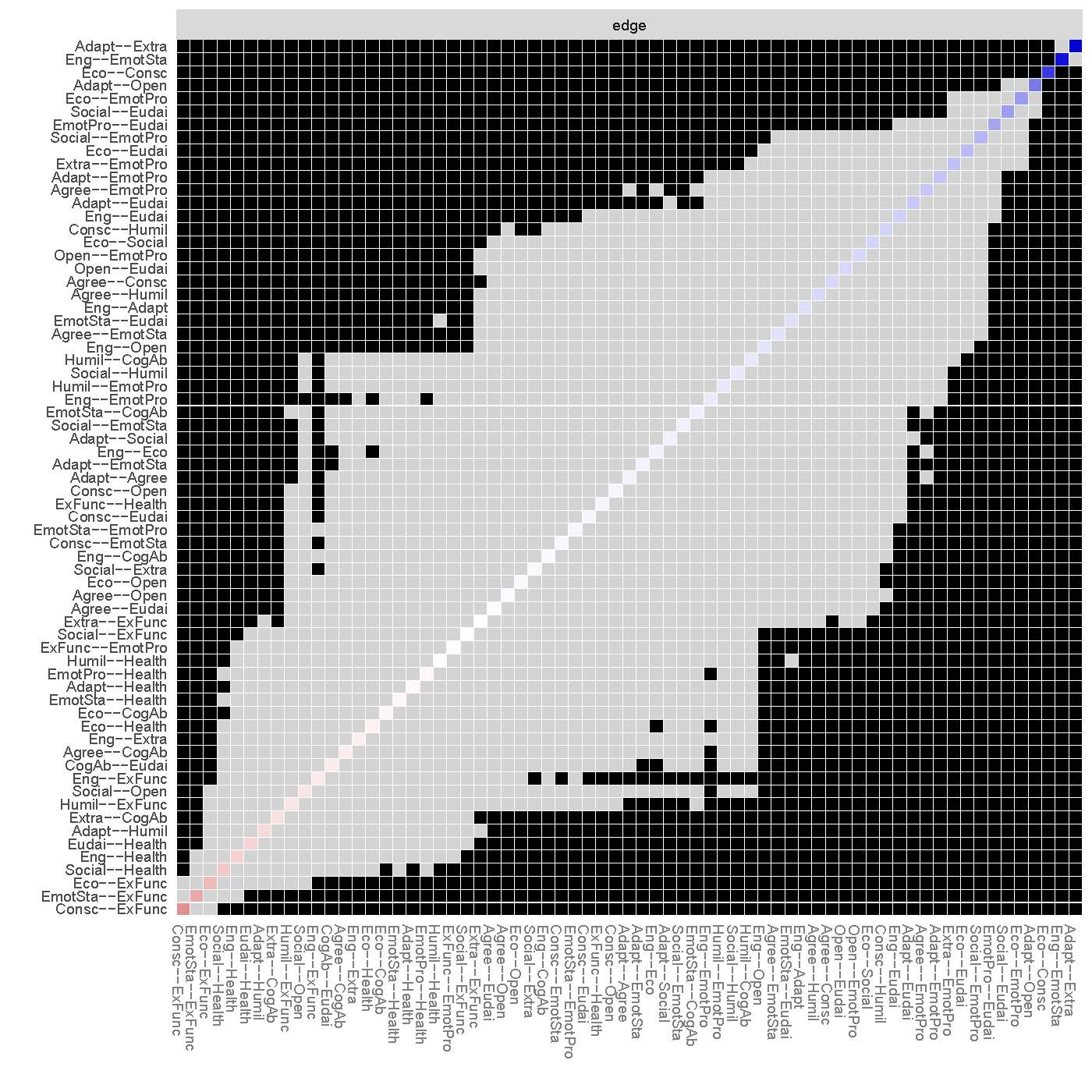 |
| --- | --- |
| Time 1 (Lower Disturbance) | Time 1 (Higher Disturbance) |
| 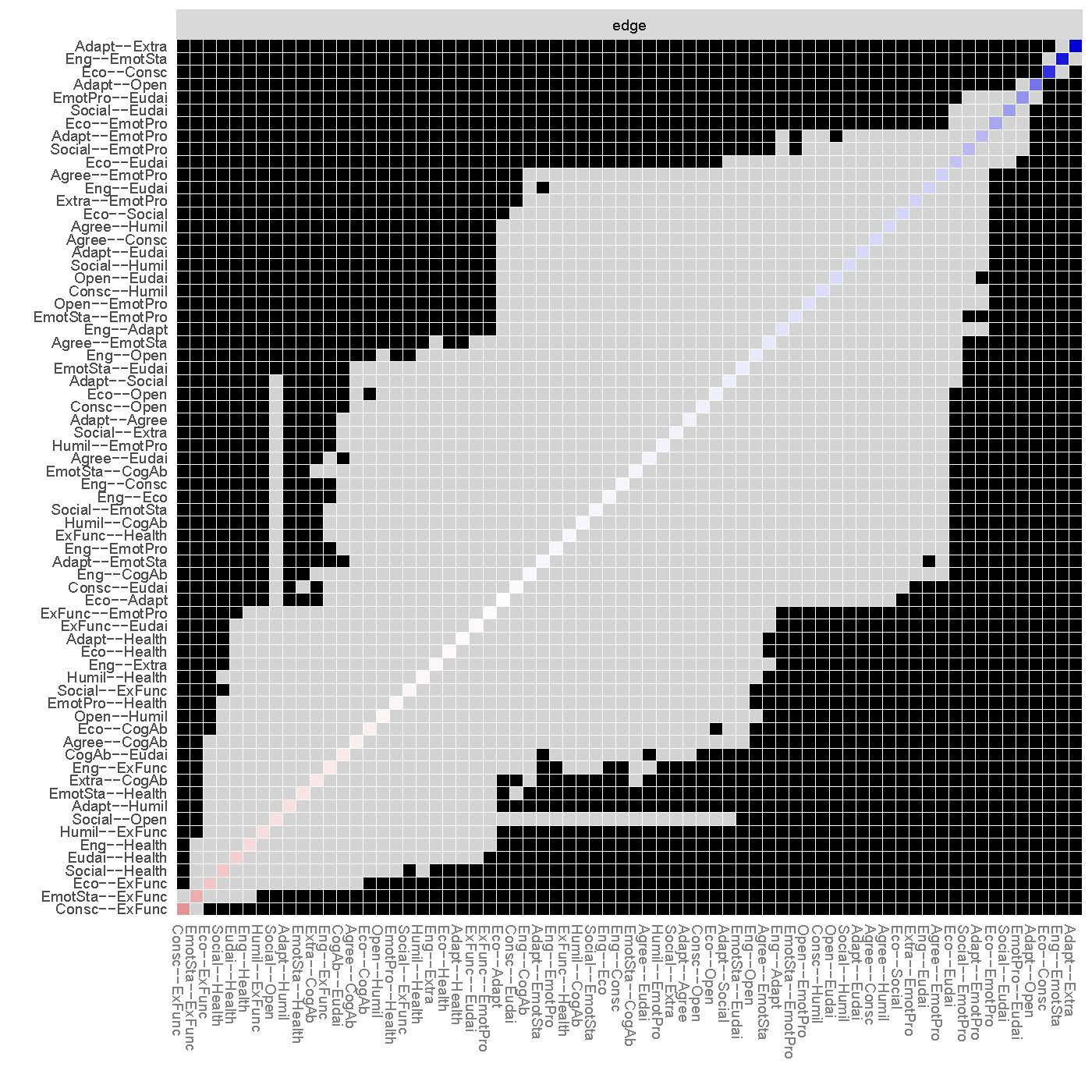 | 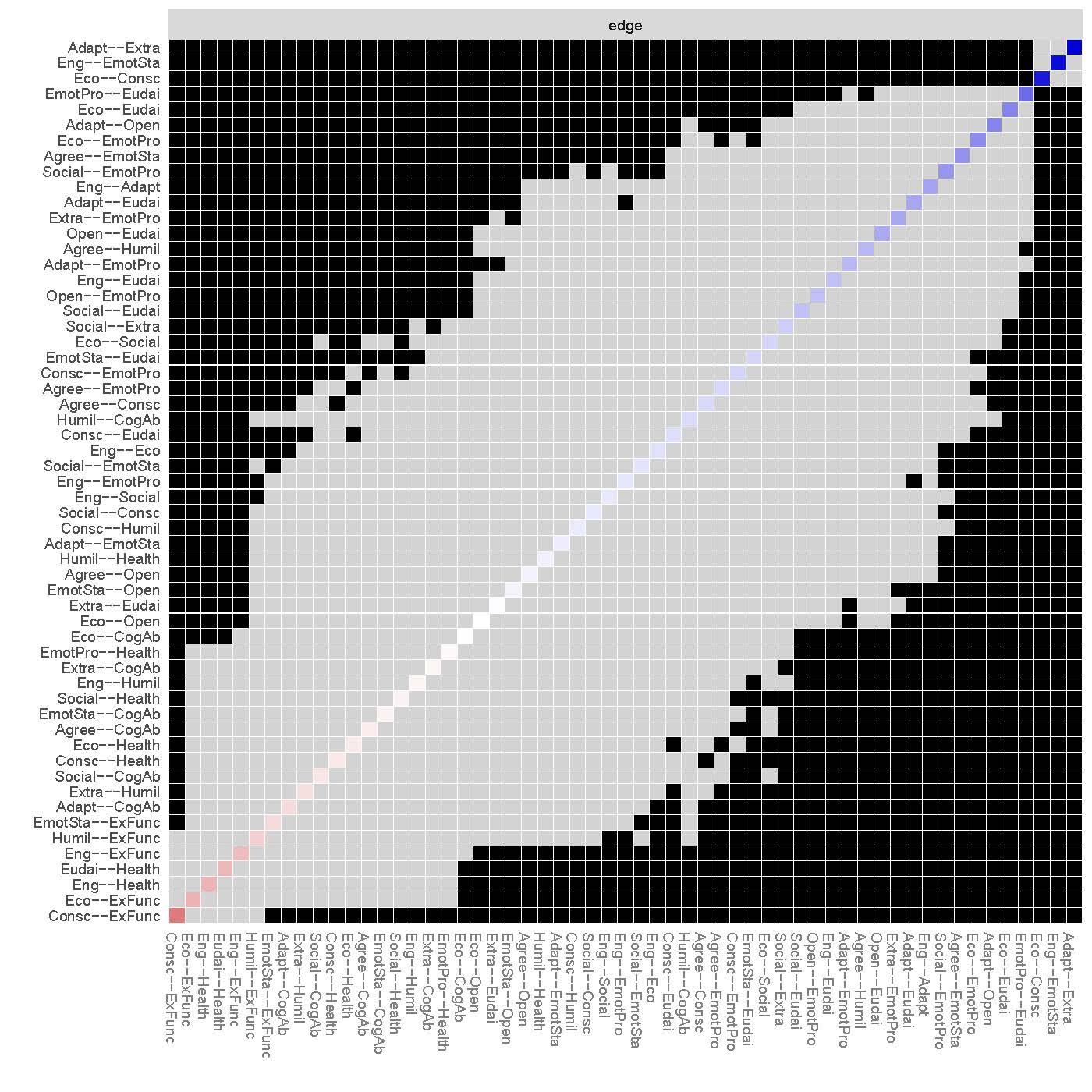 |
| Time 2 (Lower Disturbance) | Time 2 (Higher Disturbance) |

Note: Blue boxes correspond to positive correlations, while red boxes indicate negative correlations. Grey boxes indicate that the edge weights do not significantly differ (*p*>.05). Black boxes represent significant difference between the edge weight (*p*<.05).

Figure S9

*Bootstrapped edge weights difference test for the trait resilience systems network by Time by Disturbance status.*

| 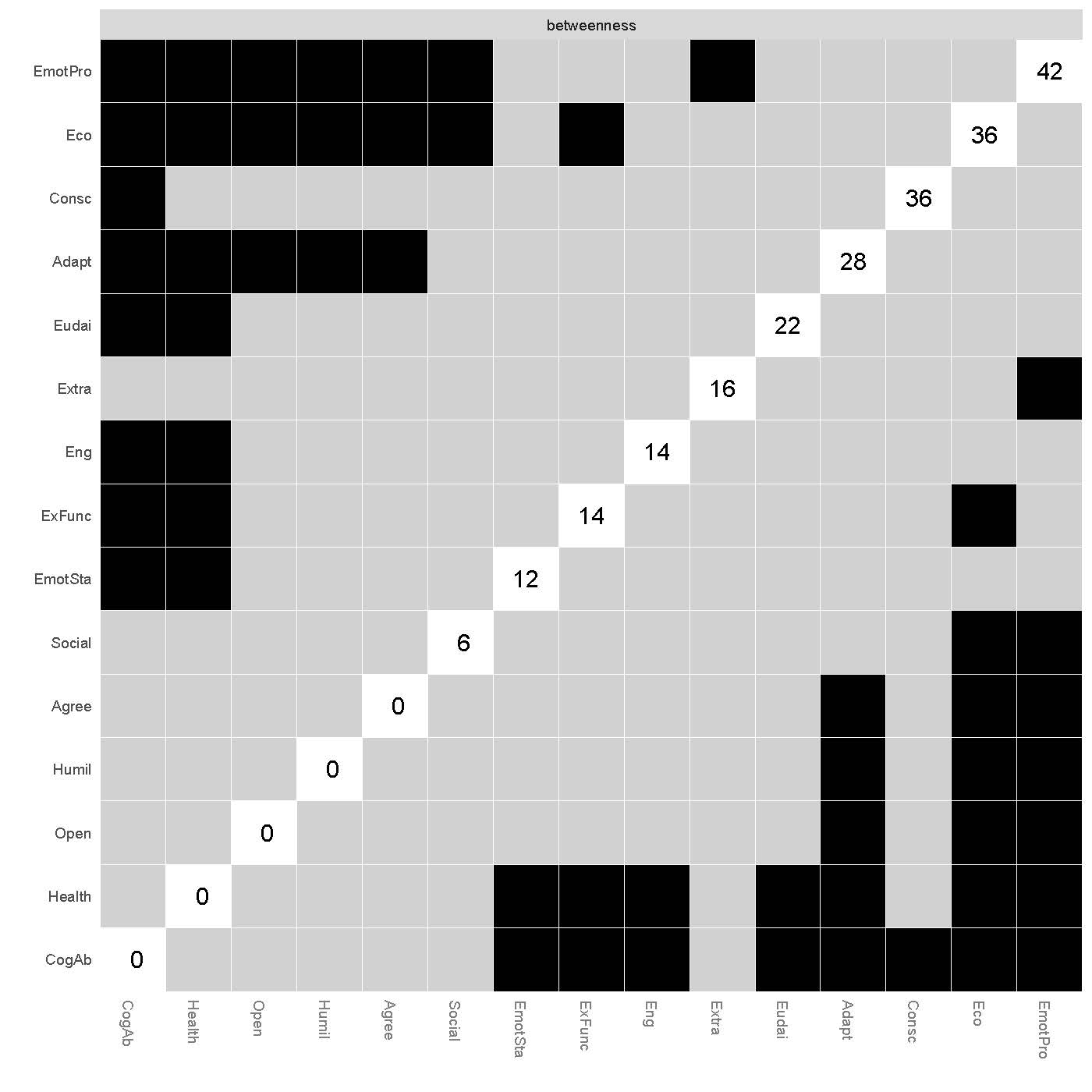 | 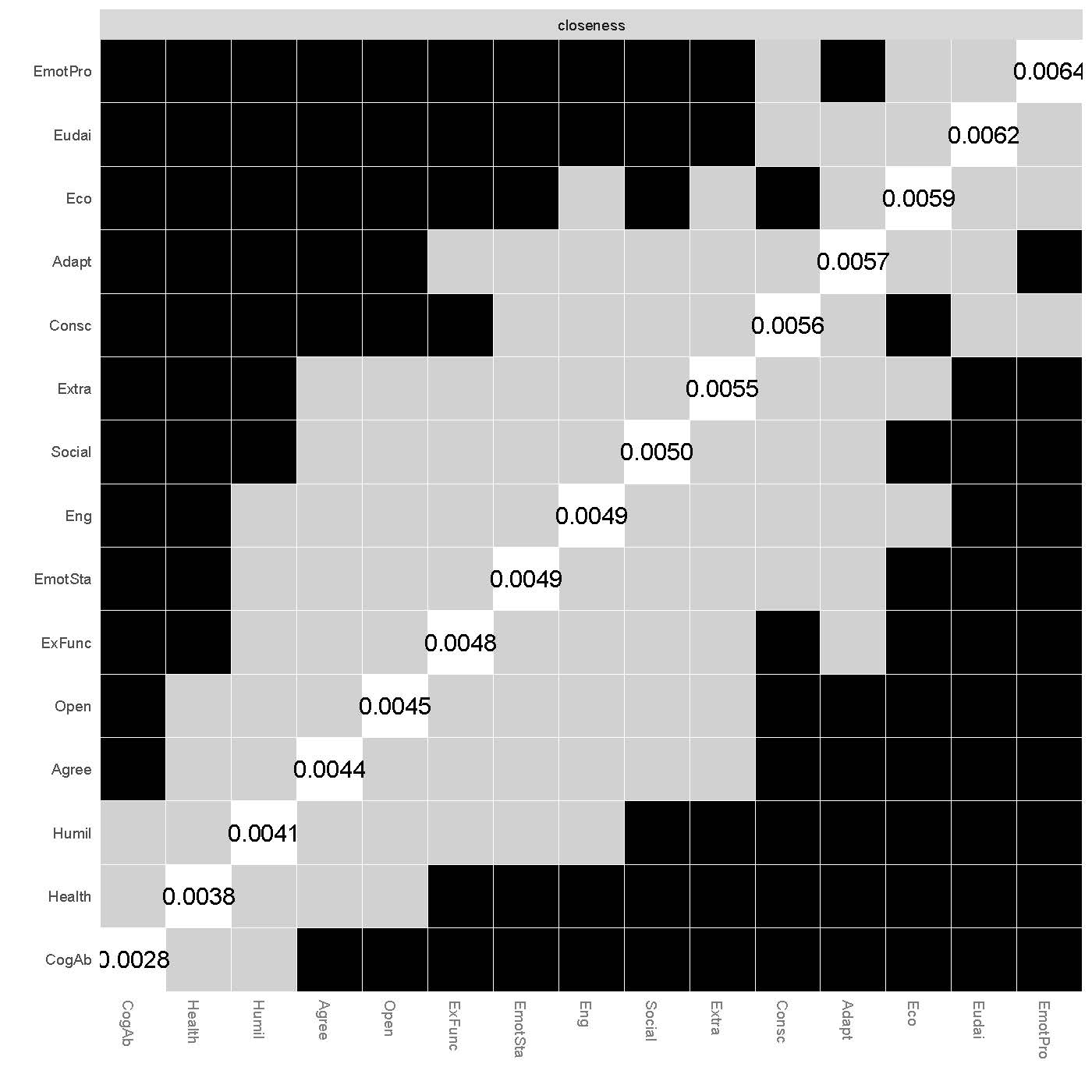 |
| --- | --- |
| Time 1 (Lower Disturbance) | |
| 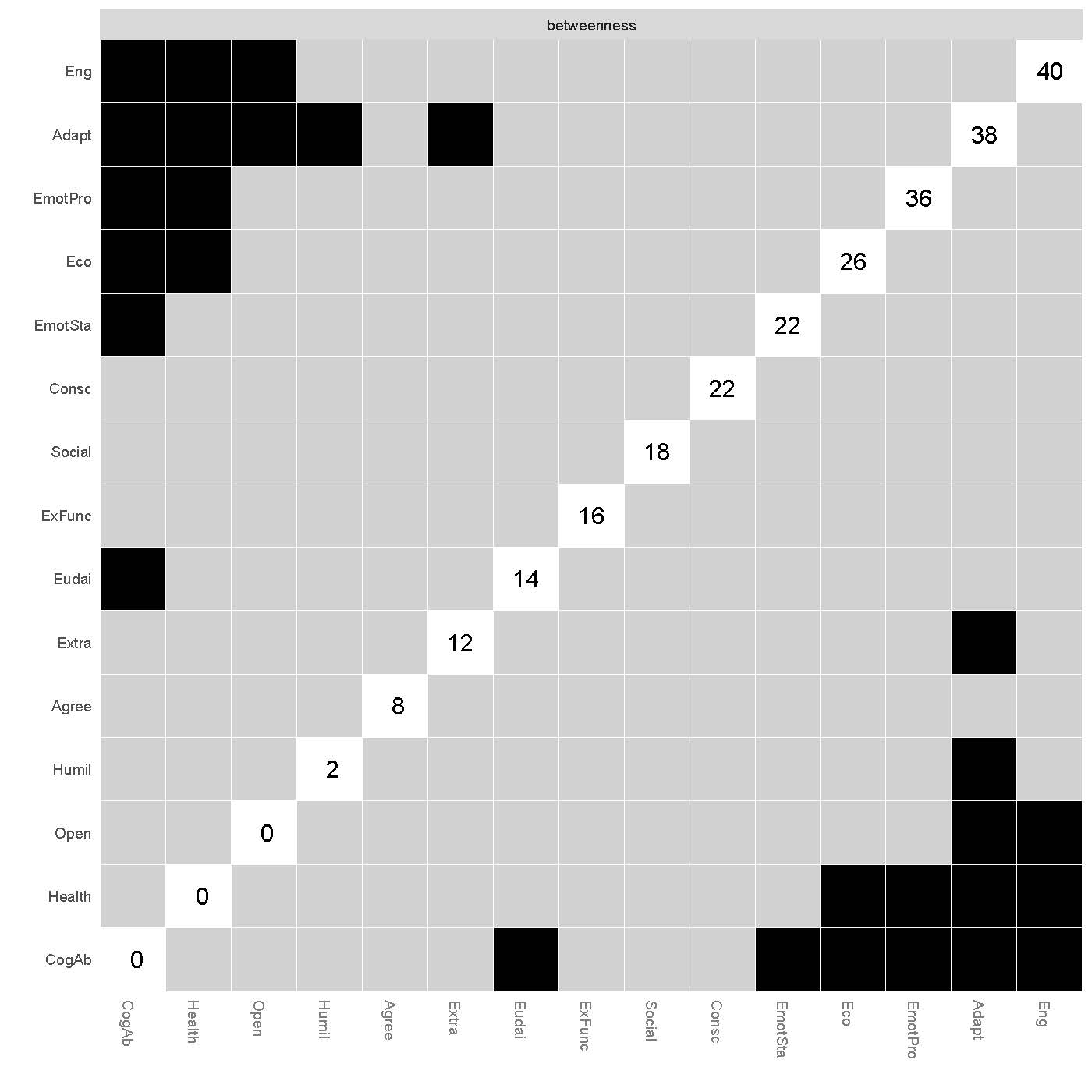 | 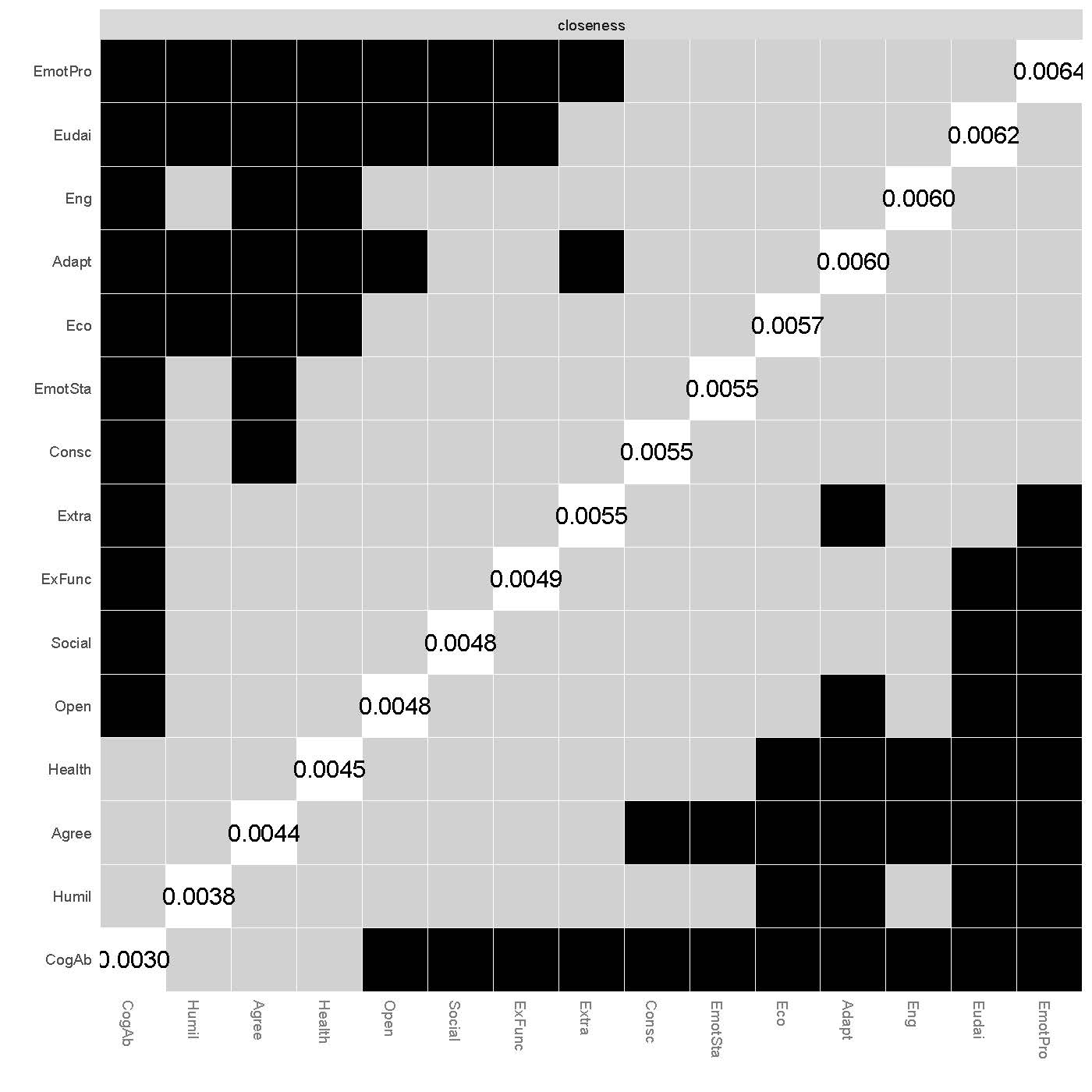 |
| Time 1 (Higher Disturbance) | |
| 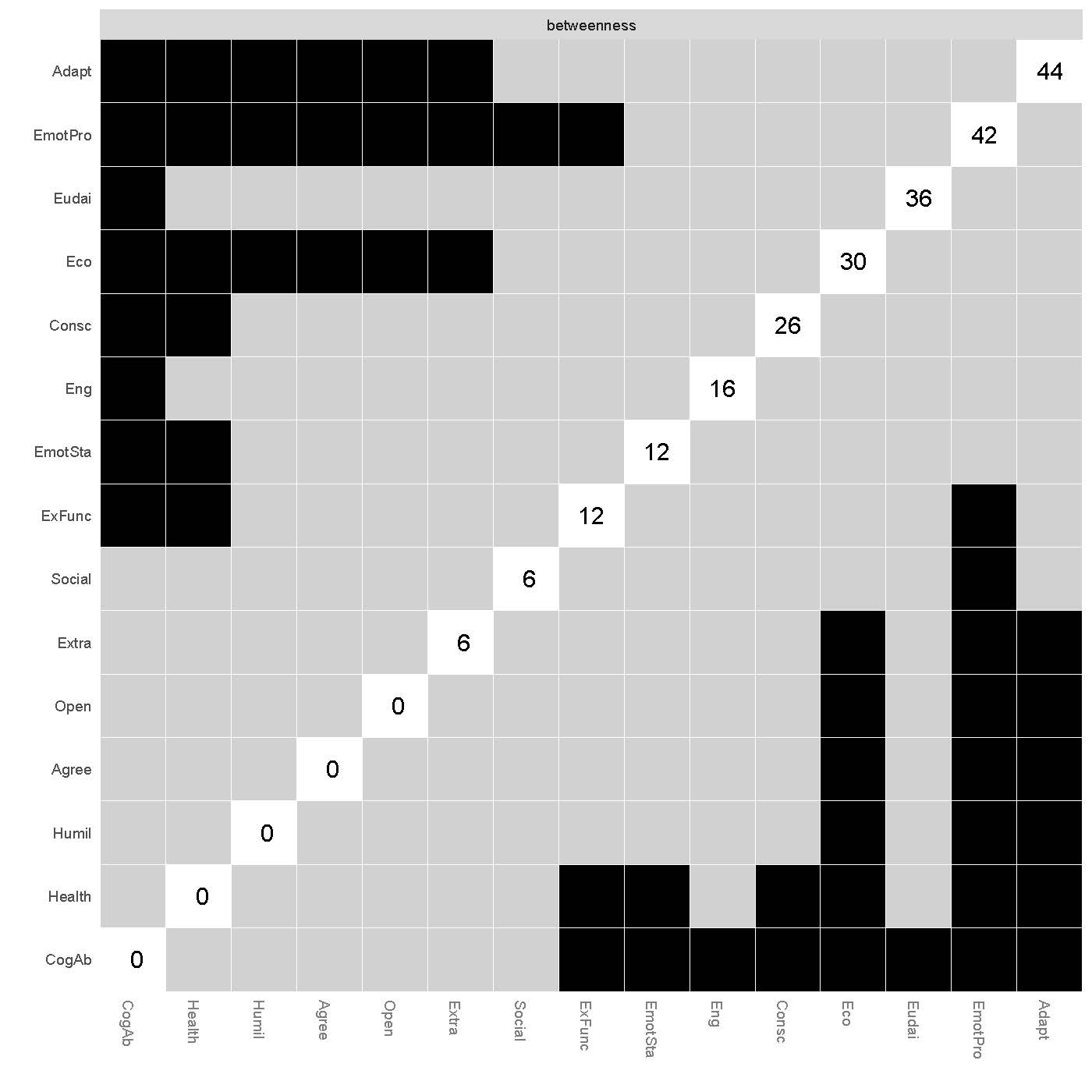 | 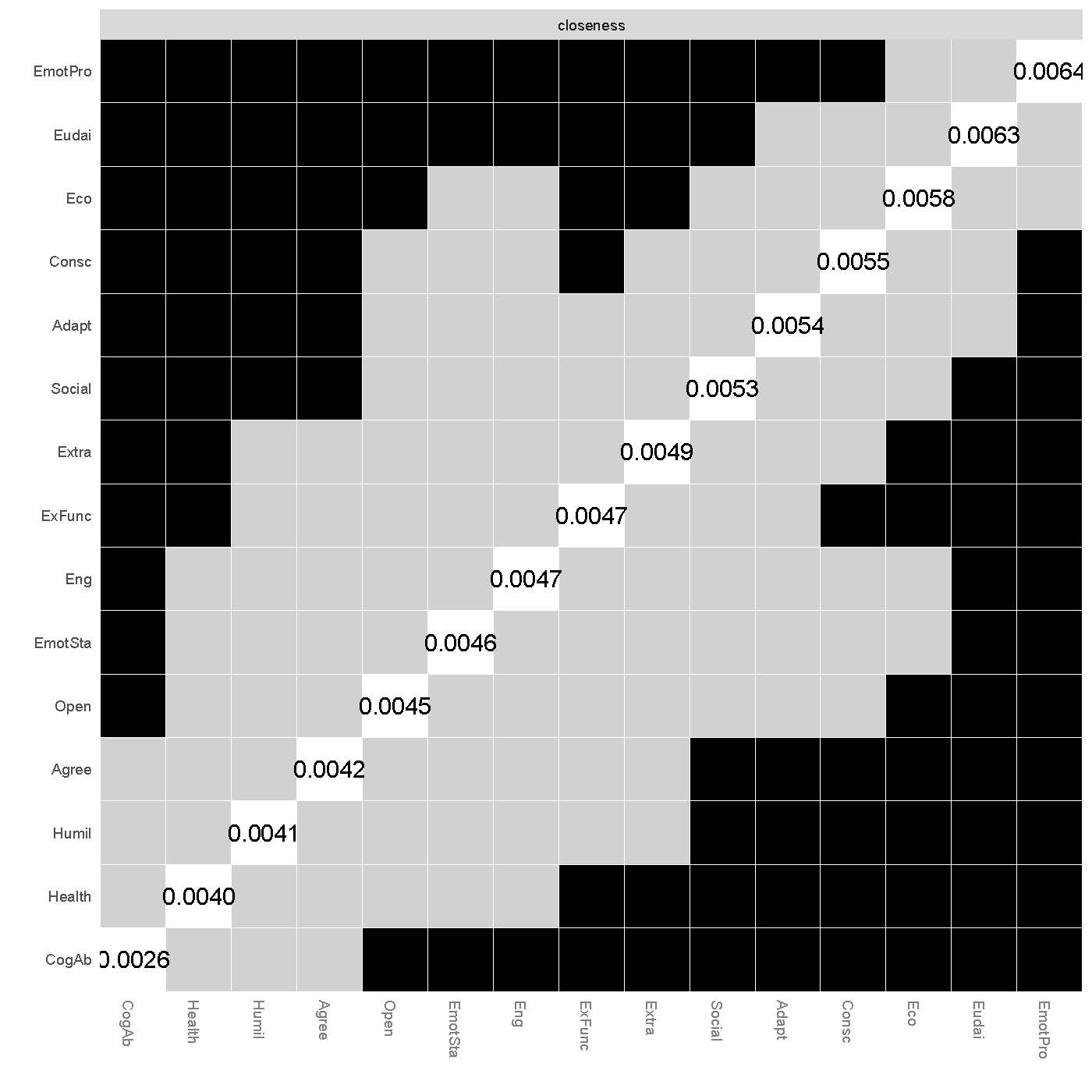 |
| Time 2 (Lower Disturbance) | |
| 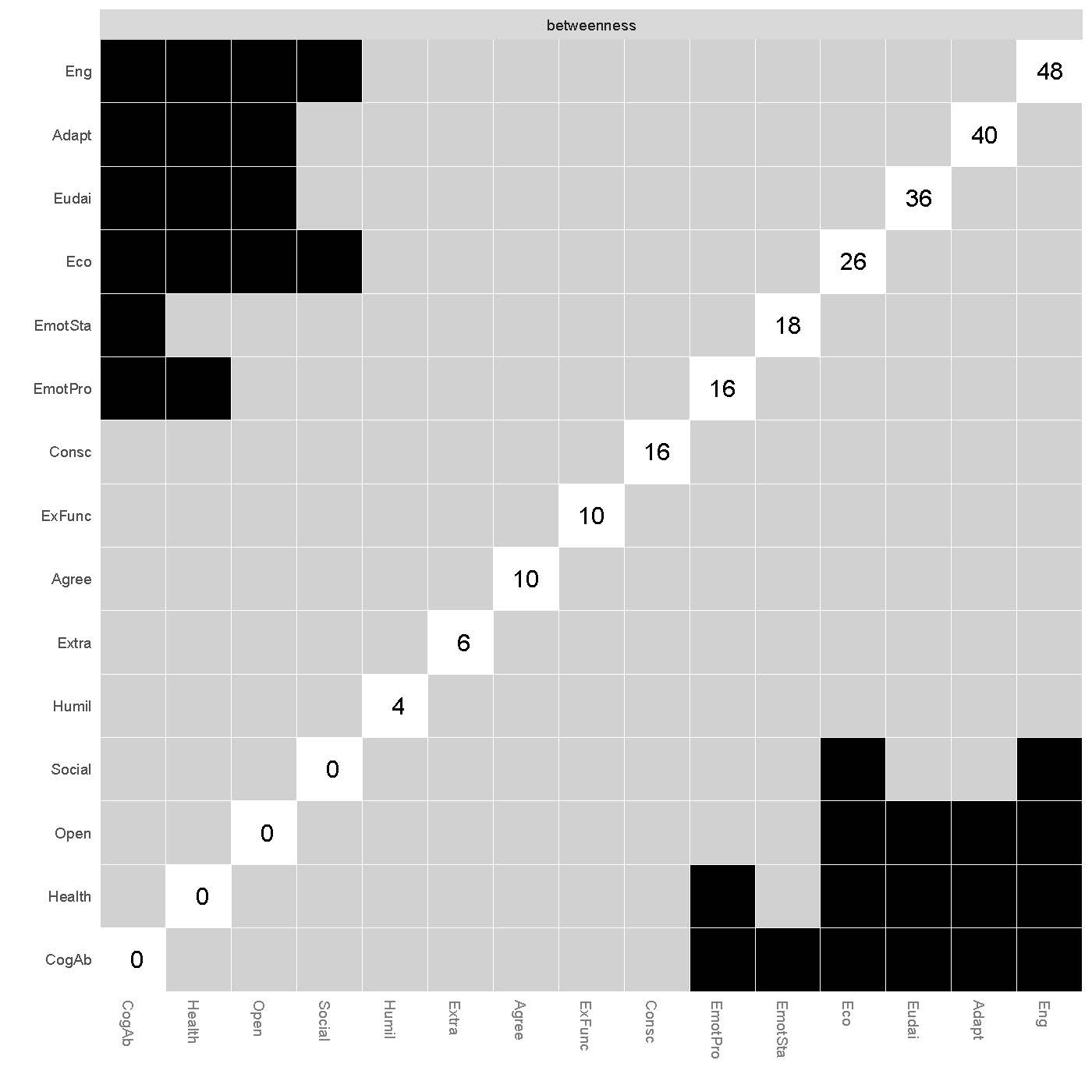 | 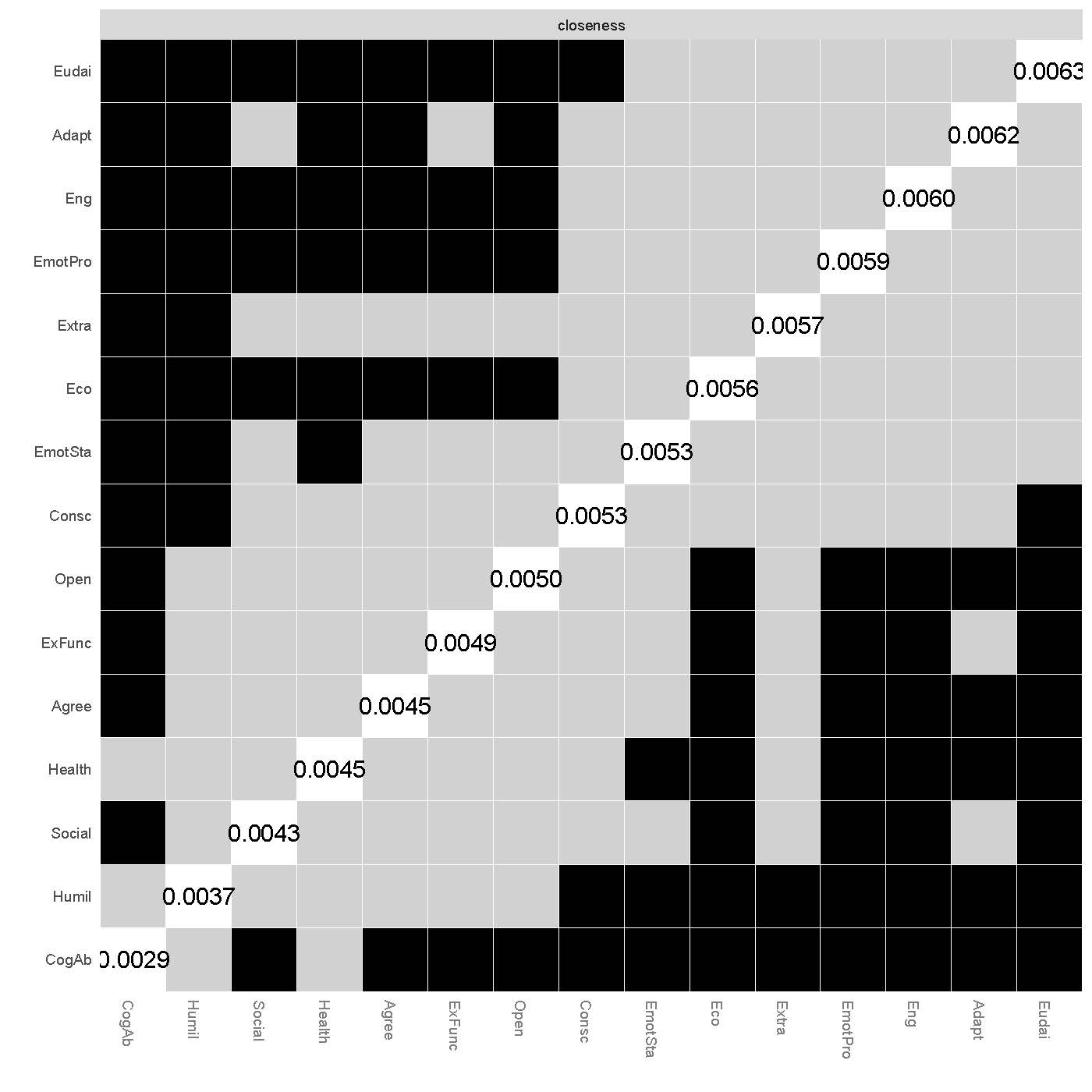 |
| Time 2 (Higher Disturbance) | |

*Note*: This test presents the bootstrapped significance of betweenness and closeness centrality estimates for each pairwise node comparison for the trait resilience systems network White boxes represent the values of node strength. Gray boxes indicate that the nodes do not significantly differ (*p*>.05). Black boxes represent significant difference (*p*<.05).

Supplementary Material S11

*Bootstrapped centrality difference test for betweenness and closeness nodes by Time for Disturbance*
